# Generation and physiological characterization of genome-edited *Nicotiana benthamiana* plants containing zeaxanthin as the only leaf xanthophyll

**DOI:** 10.1007/s00425-023-04248-3

**Published:** 2023-10-05

**Authors:** Maria Sulli, Luca Dall’Osto, Paola Ferrante, Zeno Guardini, Rodrigo Lionel Gomez, Paola Mini, Olivia Costantina Demurtas, Giuseppe Aprea, Alessandro Nicolia, Roberto Bassi, Giovanni Giuliano

**Affiliations:** 1 Casaccia Research Centre, Biotechnology and Agro-Industry Division, Italian National Agency for New Technologies, Energy and Sustainable Development (ENEA), Via Anguillarese 301, 00123 Rome, Italy; 2 Biotechnology Department, University of Verona, Strada Le Grazie 15, 37134 Verona, Italy; 3 Council for Agricultural Research and Economics, Research Centre for Vegetable and Ornamental Crops (CREA), Via Cavalleggeri 25, 84098 Pontecagnano, Italy; 4 Present Address: Facultad de Ciencias Agrarias, Universidad Nacional de Rosario (UNR), Campo Experimental Villarino CC No 14, Zavalla – Santa Fe, Argentina

**Keywords:** Zeaxanthin, Zeaxanthin epoxidase, Lycopene epsilon cyclase, Abscisic acid, Photosynthetic apparatus, Photosynthesis, LHCII, NPQ, Photoprotection, Genome editing

## Abstract

**Main conclusion:**

**Simultaneous genome editing of the two homeologous** ***LCYe*** **and** ***ZEP*** **genes of** ***Nicotiana benthamiana*** **results in plants in which all xanthophylls are replaced by zeaxanthin**.

**Abstract:**

Plant carotenoids act both as photoreceptors and photoprotectants in photosynthesis and as precursors of apocarotenoids, which include signaling molecules such as abscisic acid (ABA). As dietary components, the xanthophylls lutein and zeaxanthin have photoprotective functions in the human macula. We developed transient and stable combinatorial genome editing methods, followed by direct LC–MS screening for zeaxanthin accumulation, for the simultaneous genome editing of the two homeologous *Lycopene Epsilon Cyclase* (*LCYe*) and the two *Zeaxanthin Epoxidase* (*ZEP*) genes present in the allopolyploid *Nicotiana benthamiana* genome. Editing of the four genes resulted in plants in which all leaf xanthophylls were substituted by zeaxanthin, but with different ABA levels and growth habits, depending on the severity of the *ZEP1* mutation. In high-zeaxanthin lines, the abundance of the major photosystem II antenna LHCII was reduced with respect to wild-type plants and the LHCII trimeric state became unstable upon thylakoid solubilization. Consistent with the depletion in LHCII, edited plants underwent a compensatory increase in PSII/PSI ratios and a loss of the large-size PSII supercomplexes, while the level of PSI-LHCI supercomplex was unaffected. Reduced activity of the photoprotective mechanism NPQ was shown in high-zeaxanthin plants, while PSII photoinhibition was similar for all genotypes upon exposure to excess light, consistent with the antioxidant and photoprotective role of zeaxanthin in vivo.

**Supplementary Information:**

The online version contains supplementary material available at 10.1007/s00425-023-04248-3.

## Introduction

Carotenoids are essential components of the plant photosynthetic apparatus, whose roles in photosystem assembly and photoprotection have been largely investigated (Dall’Osto et al. 2007; Havaux et al. 2007, 2004). Plant Photosystem I (PSI) and II (PSII) contain xanthophylls like lutein, violaxanthin, zeaxanthin and neoxanthin in the peripheral light-harvesting complexes, while β-carotene is localized in PSII core complexes, and in both PSI peripheral antenna and core complex (Wei et al. 2016; Qin et al. 2015).

The higher plant photosynthetic apparatus exhibits a high degree of plasticity with respect to the carotenoid species it can functionally accommodate. Plants containing zeaxanthin or astaxanthin as the only xanthophyll (Havaux et al. 2004; Xu et al. 2020), or lacking lutein (Dall'Osto et al. 2006) have been described and characterized photosynthetically.

**Fig. 1:**
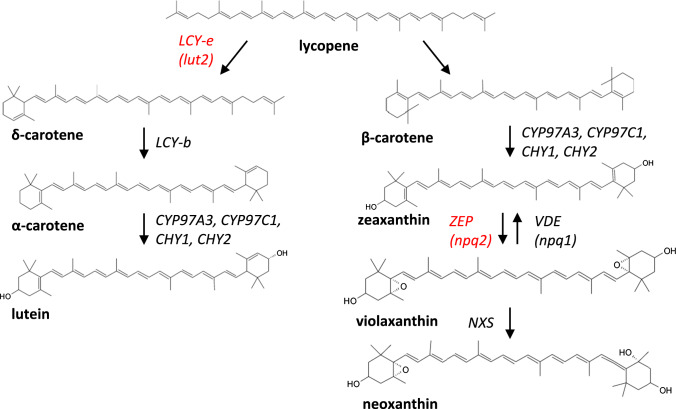
Schematic carotenoid biosynthetic pathway. Genes targeted for editing are indicated in red. Names between parentheses refer to the *Arabidopsis* mutant nomenclature

Leaves of wild-type plants only accumulate zeaxanthin upon exposure to excess light stress from de-epoxidation of pre-accumulated violaxanthin (Fig. 1). The photo-protective effect of zeaxanthin is exerted both through increased thermal dissipation of excess energy, and through the protection of thylakoid membranes from peroxidation (Johnson et al. 2007). As described based on phenotypes of zeaxanthin deficient *Arabidopsis* mutants such as *npq1 lut2*, which showed a decreased capacity for photoprotection in excess light (Niyogi et al. 2001), as compared to *npq2 lut2*, with all xanthophyll sites occupied by zeaxanthin, showing higher resistance to photo-oxidative stress (Havaux et al. 2004).

In humans, dietary lutein and zeaxanthin also play a photo-protective role and are accumulated in several organs, including the macula lutea, in the central portion of the retina. Age-Related-Macular Degeneration (AMD), representing the main cause of blindness in elderly people, is inversely correlated with zeaxanthin and lutein intake (Wu et al. 2015; Richer et al. 2011; Arunkumar et al. 2018). Several additional health-promoting effects of zeaxanthin, including reduced risk of atherosclerosis and breast cancer, have been described (Voutilainen et al. 2006; Johnson 2014; Bernstein et al. 2016). While green leafy vegetables are rich in lutein, the natural sources of zeaxanthin are much more limited (Karniel et al. 2020). Hence, there is an interest in engineering new plant sources of zeaxanthin.

*Nicotiana benthamiana* is a model system for plant transformation, as well as for the application of new plant breeding techniques such as CRISPR/Cas9-mediated genome editing, transient expression through agroinfiltration or virus-mediated overexpression, and virus-induced gene silencing. Because of its amenability to growth in contained conditions—ideal for the production of biopharmaceuticals—and its status as a non-food crop, *N. benthamiana* is a choice platform for the production of biopharmaceuticals. Several studies described the genome editing of *N. benthamiana* to accumulate modified endogenous metabolite levels with agronomic, industrial, nutritional and health-related value (Molina-Hidalgo et al. 2021). However, its 3.2 Gb genome is the result of an allopolyploidization that occurred ~ 6 mya. As a result, about 50% of the genes that are found in single copy in other plants, are duplicated in *N. benthamiana* (Ranawaka et al. 2023), increasing the difficulty of knocking out biosynthetic steps that are encoded by still-duplicated genes.

Here, we report methods for the transient and stable combinatorial transformation of *N. benthamiana* leaves with *A. tumefaciens*, for (i) optimizing genome editing constructs and (ii) using them for the simultaneous editing of the two *Lycopene Epsilon Cyclase* (*LCYe*) and two *Zeaxanthin Epoxidase* (*ZEP*) genes, to generate high-zeaxanthin (HZ) plants containing zeaxanthin as the only leaf xanthophyll. Two phenotypically different types of HZ mutants were obtained, HZ-9 and HZ-11. Both mutants contained zeaxanthin as the only leaf xanthophyll and much lower ABA levels than wild-type (WT), with HZ-11 exhibiting a more severe ABA-deficient phenotype.

Replacement of the normal leaf xanthophyll complement by zeaxanthin only, reduced accumulation of the major trimeric antenna complex (LHCII) by 50% with respect to WT and triggered a compensatory increase in PSII/PSI ratio (+ 60%). Native electrophoresis highlighted a reduced stability of trimeric LHCII, which dissociates into its monomeric components, and a lower abundance of PSII supercomplex high molecular weight (MW) bands, while the stability of PSI-LHCI supercomplex was essentially unaffected. HZ plants underwent a reduced activity of ΔpH-regulated thermal energy dissipation (NPQ activity was reduced by 50% with respect to WT at irradiances > 300 µmol photons m^−2^ s^−1^), suggesting plants might suffer for enhanced photoinhibition under photoxidative conditions. Instead, constitutive accumulation of zeaxanthin did not hamper phototolerance respect to WT plants, despite the depletion of two crucial photoprotective components, namely NPQ and trimeric LHCII. These results indicate that the capacity of zeaxanthin to protect photosynthetic apparatus is significantly higher than that of all other xanthophylls.

## Materials and methods

### Plant materials and growth conditions

*Nicotiana benthamiana* (LAB) plants were grown under controlled photoperiod (16 h light/8 h dark, 150 µmol photons m^−2^ s^−1^) and temperature (24 °C) conditions. For carotenoid and ABA analyses, three leaves for each line were sampled 5 weeks after germination, frozen in liquid nitrogen and freeze-dried. For photosynthetic analyses, plants were grown in soil, in a phytotron for 6 weeks at 150 μmol photons m^−2^ s^−1^, 8 h light/16 h dark, 23 °C, 70% relative humidity. All biochemical and physiological analyses were carried out on plants prior to the initiation of flowering.

### Design and optimization of CRISPR-CAS9 editing vectors

sgRNAs were designed for the simultaneous targeting of each homeologous gene pair. The CRISPR-P tool (http://crispr.hzau.edu.cn/cgi-bin/CRISPR2/CRISPR) was used to build the RNA guides and determine the corresponding off-target, using *N. benthamiana* v. 0.4.4 as the target genome, and the sgRNAs were validated also with *N. benthamiana* v. 0.5 (http://www.benthgenome.qut.edu.au/) (Gene IDs are provided in File S1). Two sgRNAs for each gene (4 in total, Fig. S1a) were cloned in the pDGB3-α1 vector under the U6-26 promoter using Goldenbraid multipartite assembly (Vazquez-Vilar et al. 2016), and the editing efficiency was tested through combinatorial agroinfiltration of *N. benthamiana* leaves together with a third construct containing either a 35S:HsCAS9:NOS or an UBI:AtCAS9:RBCS transgene (Fig. S1b; vector maps and sequences are provided in Files S2–5). Each construct was transformed into *A. tumefaciens* GV3101 strain and combinatorial agroinfiltration of 4 weeks-old *N. benthamiana* plants was carried out according to the protocol of (Wood et al. 2009) using different CAS9 and sgRNA plasmids. Six days later, total DNA was extracted from agroinfiltrated leaves using DNeasy Plant Mini Kit (Qiagen – Hilden, Germany) according to manufacturer instructions. sgRNA target regions were amplified from 50 ng of genomic DNA with Q5 High-Fidelity DNA Polymerase (NEB, Ipswich, Massachusetts, USA) using the primers pairs: LCYe1 _T4_For/LCYe2_T4_For and LCYe_T4_Rev, for both the Lcye_T4 and Lcye_T5 targets; ZEP1_T1_FOR/ZEP2_T1_FOR and ZEP_T1_REV, for the ZEP_T1 target; ZEP1_T3_FOR/ZEP2_T3_FOR and ZEP_T3_REV, for the ZEP_T3_target (Table S1). Off-target amplicons were generated similarly (Table S1). All the primers for target and off-target analysis (Merck, Darmstadt, Germany) included the Illumina (San Diego, California, USA) standard overhang adapter sequences at 5’ end. PCR amplicons were purified with the Agencourt AMPure XP PCR purification (Beckman Coulter, Brea, California, USA) and sequenced by NGS (2 × 250 paired end, 100 × coverage). The resulting amplicon pools were analyzed with the software CRISPResso (http://crispresso2.pinellolab.org/submission; (Clement et al. 2019)), to identify and estimate the frequency of indels in target and off-target sequences (Table S2).

### *N. benthamiana* combinatorial transformation

The pDGB3-α1- LCYe_T5, DGB3-α1- ZEP-T1 and pAtCas9-NPTII vectors were used to transform *Agrobacterium* strain LBA4404 and glycerol stocks were obtained individually for each vector. The protocol followed for *Agrobacterium* growth conditions was as described (Mini et al. 2018), for plant transformation and in vitro conditions (Tavazza et al. 1988). In detail, glycerol stocks carrying the three vectors were inoculated in 10 ml liquid YEB medium containing 30 μg/ml rifampicin and 100 μg/ml kanamycin and grown at 28 °C for 24 h at 130 rpm. Then the cultures were diluted 1:100 in 10 ml of AB minimal medium pH 7 supplemented with 30 μg/ml rifampicin and 100 μg/ml kanamycin and grown for 24 h at 28 °C. The cultures were centrifuged at 2500 rcf for 15 min at room temperature, resuspended in a final volume of 20 ml AB induction medium pH 5.6 without antibiotics to an OD of 0.2 and then grown for 1 h to a final OD of 0.3. The three cultures were then mixed and 20 ml were placed in a petri dish containing about 50 leaf pieces for each transformation. The plate was gently mixed for 20 min and the leaf pieces were blotted dry on sterile filter paper and placed on growth media according to Tavazza et al. (1988).

### Genomic DNA isolation

Genomic DNA was isolated from two leaf disks of *N. benthamiana* plants obtained from stable *Agrobacterium* transformation following a standard phenol–chloroform protocol. Briefly, two young leaf disks from each plant were collected in 2 ml Eppendorf tubes and disrupted using a 3 mm tungsten carbide bead (Qiagen Cat. No. 69997) in a Tissuelyser (1 min, maximum frequency). Eight hundred µl of extraction buffer (Tris–HCl 200 mM pH 7.5, NaCl 250 mM, EDTA 25 mM, SDS 0.5%, β-mercaptoethanol 0.1%) and 200 µl of Phenol:Chloroform:Isoamyl Alcohol 25:24:1, saturated with 10 mM Tris, pH 8.0, 1 mM EDTA were added to each sample and homogenized using a Tissuelyser for 1 min at maximum frequency. The samples were centrifuged at 13,000 rpm for 10 min and the supernatant (about 600 µl) was recovered in a new 1.5 Eppendorf tube. DNA was precipitated by adding 500 µl of isopropanol and keeping the tubes for 10 min on ice. Samples were then centrifuged at 13,000 rpm for 10 min and the supernatant was carefully removed and discarded. DNA pellets were washed with 400 µl of EtOH 80% and centrifuging at 13.000 rpm for 10 min. After removing the supernatant, DNA pellets were air dried for 5 min at room temperature and then resuspended with 100 µl of TE, supplemented with 100 ng/µl RNAse A (Merck Cat. No. R6513). The samples were incubated at 37 °C for 30 min to digest RNA and 2 µl of each sample was used for each PCR amplification reaction. All the centrifugation steps were made at room temperature.

### PCR genotyping and ICE analysis

Genomic DNA from T_0_ plants was analyzed by PCR for the presence of the three plasmids pDGB3-α 1-LCYe-T5, pDGB3-α1-ZEP-T1 and pAtCas9-NPTII using the following primers listed in Table S1: U626 _FOR 1323 and LCY_sgRNA_1497 for pDGB3-α 1-LCYe-T5, U626 _FOR 1323 and ZEP_sgRNA_1502 for pDGB3-α1-ZEP-T1, CAS9 FOR 7141 and CAS9 REV 7416 for pAtCas9-NPTII. Primers Nb_LCY1_FOR 1875 and Nb_LCY1_REV 2113 were used as positive amplification controls. Seeds of *T*_0_ plants containing all three vectors were grown and T_1_ plants analyzed by PCR for Cas9 segregation and for the presence of indels in the target *LCYe* and *ZEP* genes. Amplicons containing the target editing sites were obtained amplifying genomic DNA with primers discriminating between the two homeologous *LCYe* and *ZEP* genes (Table S1): NbLCY1 _T5For and Nb + Nt LCY_T5Rev (*LCYe1*), NbLCY2_T5 For and Nb + Nt LC T5Rev (*LCYe2*), Nb ZEP1 for_seq and Nb ZEP_ rev_seq (*ZEP1*), Nb ZEP2 for_seq and Nb ZEP_ rev_seq (*ZEP2*). All PCR reactions were performed using 2 µl of genomic DNA and Dream Taq polymerase (Thermo Scientific Cat. No. EP0702). PCR were analyzed by capillary electrophoresis using the Qiaxcel Advanced instrument (Qiagen) and QX DNA Screening kit (Qiagen Cat. No. 1050349). The four amplicons (*LCYe1*, *LCYe2*, *ZEP1*, *ZEP2*) containing the target editing sites were treated with EXOSAP-IT (Applied Biosystems, Cat. No. 78201.1), analyzed by Sanger sequencing through the online software “ICE CRISPR analysis tool” from Synthego (https://www.sthego.com/products/bioinformatics/crispr-analysis). Genes with a knockout (KO) score > 90 were considered edited.

### LC–MS screening of T_1_ edited plants

3 mg of homogeneously ground freeze-dried leaf tissue x each sample was used for carotenoid extraction, performed as previously reported (Fantini et al. 2019). Carotenoids in leaves of T_1_ plants were analyzed using Liquid Chromatography- High-Resolution Mass Spectrometry, LC-HRMS system, using a Dionex Ultimate HPLC (Dionex) coupled to a Q-Exactive Hybrid Quadrupole-Orbitrap Mass Spectrometer (Thermo Fisher Scientific). Ionization was obtained by Atmospheric Pressure Chemical Ionization (APCI) source, operating in positive and negative ionization mode. LC separations were performed using the YMC Carotenoid C30 column (100 × 3 mm, 3-µm particle size; YMC; Europe GmbH, Dinslaken, Germany), solvent system and MS settings were used as previously reported (Fantini et al. 2019). Total run time was 18 min and the column temperature was 25 °C. Masses were detected in the 110–1100 m/z range. Relative levels of accumulation of carotenoids were calculated as fold of integrated areas under the m/z peak of the preferred ion of each carotenoid and the internal standard peak area (Fold-IS), using the Xcalibur Quan Tool (Thermo Fisher Scientific, Cambridge, MA, USA). Chemicals and solvents were LC–MS grade quality (CHROMASOLV^®^, from Merck group KGaA, Darmstadt, Germany).

### LC-PDA quantification of carotenoids

Extraction procedure and saponification were performed from 10 mg of ground, freeze-dried leaf tissue according to the recently described protocol (Demurtas et al. 2023). LC separation was performed using an LC-PhotoDiode Array (PDA) system (Accela, Thermo Fisher Scientific, Thermo Fisher Scientific, Waltham, MA, USWaltham, MA, US) and a carotenoid C30 column (100 × 3 mm^2^, 3-µm particle size) (YMC, Dinslaken, Germany), the column temperature was 25 °C. Elution system was A, MeOH; B, MeOH/water (4:1 *v*/*v*) with 0.2% ammonium acetate; and C, tert-Butyl methyl ether (C), and gradient was 0 to 1.2 min 95% A, 5% B; 3.5 min 80% A, 5% B; 6.8 min 30% A, 5% B, 65% C; 16 min 95%, 5%. Injection volume was 10 µL, chromatographic flux after equilibration was 0.8 ml/min and the total run time 18 min. PDA data were recorded in the 200–700 nm range. Carotenoids were measured based on their absorption spectra as detected by the PDA, integrated at their individual λmax and normalized to DL-α-tocopherol acetate (Sigma–Aldrich, Cat. No. T3376-5G) at 285 nm, as recently described (Demurtas et al. 2023).

### LC–MS quantification of abscisic acid

For ABA analysis, leaves were subjected as previously described (Frusciante et al. 2022) with some modifications. Briefly, 10 mg of freeze-dried leaf powder was resuspended in 750 µL of 75% (v/v) cold methanol spiked with 0.5 µg/mL formononetin (Sigma–Aldrich, Cat. No. 47752-25MG-F). Semi-polar metabolites were extracted by vigorous agitation for 30 min at 25 °C. Samples were then centrifuged at 20,000 rcf for 20 min, the supernatant was collected, filtered with HPLC PTFE filter tubes (0.22 µm pore size), and subjected to LC–PDA–HRMS analysis as described (Demurtas et al. 2018). ABA was quantified by LC–MS in HESI negative ionization mode, integrating the area of the M-H ion of m/z 263.1289 at the retention time of 12.10 min (normalizing to the internal standard formononetin) and using a calibration curve established for the ABA standard (Sigma–Aldrich, Cat. No. A1049-100MG).

### Genome resequencing of HZ-9 and HZ-11 mutants

Young leaves of WT, HZ-9, and HZ-11 plants were collected from one plant and freeze-dried before genomic DNA extraction using the DNeasy Plant Mini Kit (Qiagen Cat. No. 69104). DNA concentration was estimated using Quanti-it Picogreen ds DNA (Thermo Fisher Cat. No. P7589). About 1 µg of DNA was sent for sequencing with short reads (2 × 150) at 20 × coverage. For read alignment, we used a newly available chromosome-level genome assembly (Ranawaka et al. 2023) available online at https://bioweb01.qut.edu.au/benthTPM/download.html. sgRNA target and off-target loci were identified using cas-offinder v 2.4.1 (Bae et al. 2014). The target loci were confirmed as the 2 *LCYe* and the 2 *ZEP* genes. Gene identifiers for this version of the genome are indicated in Table S4 under column “target” (LCYe1: NbL16g02350; LCYe2: NbL10g02230; ZEP1: NbL12g21160; ZEP2: NbL02g05860).

We could not find any locus for 1 or 2 mismatches and we found 22 off-target loci with 3 mismatches. After cleaning the reads (fastp v 0.23.1) (Chen et al. 2018) we mapped them to the reference (BWA v 0.7.17) (Li and Durbin 2009) and called variants (gatk v 4.4.0.0 (Van der Auwera and O'Connor 2020), samtools v 1.17 (Danecek et al. 2021)), a custom script was used to identify variants on target and off-target loci, focusing on indels.

### Photosynthetic characterization

*Membrane isolation*—Stacked thylakoid membranes were isolated as previously described (Casazza et al. 2001).

*Spectroscopy*—Pigments were extracted from leaf discs with 85% acetone buffered with Na_2_CO_3_. Absorption measurements of acetone extracts were performed at RT using an SLM Aminco DW-2000 spectrophotometer. Pigments were quantified from deconvolution of spectra of acetonic extracts (Croce et al. 2002).

*Gel electrophoresis and immunoblotting*—SDS-PAGE analysis was performed using the Tris-Tricine buffer system (Schägger et al. 1988). For immunotitration, thylakoid samples (0.15–0.30–0.45 µg Chls) were loaded for each sample and electroblotted on nitrocellulose membranes. Proteins were detected with primary antibodies (Agrisera, Sweden. α-PsaA, Cat. No. AS06 172; α-CP47, Cat. No. AS04 038; α-Lhcb1, Cat. No. AS01 004; α-ATPase, Cat. No. AS05 085; α-Cytochrome *b*_6_, Cat. No. AS18 4169) and an alkaline phosphatase-conjugated secondary antibody (Sigma-Aldrich Cat. No. A3687). CP29 content in transgenic lines was quantified using an α-CP29 primary antibody made in-house (immunogen: CP29 purified from *Z. mays*) (Guardini et al. 2020). Signal amplitude was quantified using the GelPro 3.2 software (Bio-Rad). Non-denaturing Deriphat-PAGE was performed as previously described (Havaux et al. 2004). Solubilization of samples was carried out with either 0.4% or 0.8% w/v Dodecyl α-D-maltopyranoside (α-DM), at a Chl concentration of 0.5 µg/ml. In total, 35 µg of Chls were loaded in a medium-sized gel (16 cm height).

*Purification of LHC-containing fractions.* Thylakoids corresponding to 300 µg of Chls were solubilized in 600 µl of 0.8% *w*/*v* α-DM, 10 mM HEPES pH 7.5. Solubilized samples were then fractionated by ultracentrifugation (22 h at 280,000 g, 4 °C) in a 0.1–1-M sucrose gradient containing 0.06% *w*/*v* α-DM, 10 mM HEPES pH 7.5. Green bands were harvested, and pigments composition of selected fractions was analyzed from the deconvolution of spectra of acetonic extracts and by HPLC.

*Analysis of Chl fluorescence*—PSII and PSI function during photosynthesis was measured on whole leaves at RT with a DUAL-PAM-100 equipment (Walz, GmbH) according to (Baker et al. 2007). Fluorescence kinetics were measured in leaves vacuum-infiltrated with 3∙10^–5^ M 3-(3,4-dichlorophenyl)-1,1-dimethylurea (DCMU) and 100 mM sorbitol. The reciprocal of time corresponding to two-thirds of the fluorescence rise (T_2/3_) was taken as a measure of the PSII functional antenna size (Malkin et al. 1981). P700 measurements were performed as described before (Benson et al. 2015), in the dual wavelength mode (absorption at 830 and 875 nm) using a concentration of 50 µg Chls/ml in the measuring buffer (0.4 M sorbitol, 15 mM NaCl, 5 mM MgCl_2_, 10 mM HEPES–KOH pH7.5, 50 µM DCMU, 100 µM methyl viologen, 500 µM sodium ascorbate) as reported (Schiphorst et al. 2022). Traces were normalized between the minimum (beginning of each cycle) and the maximum absorption and fitted with an exponential function to determine the t_1/2_ for the antenna size calculations.

*Determination of the sensitivity to photooxidative stress—*Photooxidative stress was induced in detached leaves, exposed to excess light (2000 μmol photons m^−2^ s^−1^, 23 °C, white light provided by WH1200 PHYTO LED panel, JBEAMBIO, France) following the decay kinetics of maximal quantum yield of PSII photochemistry (F_v_/F_M_) (Havaux et al. 2004).

### Statistical analysis

For metabolomic data, ANOVA plus Tukey’s *t*-test between HZ lines and WT was performed using the Past Software (v2.17) (Hammer and Harper 2001) to determine the significant differences in carotenoid and abscisic acid contents within edited and wild-type plants (*p* ≤ 0.01), data are presented as mean values + / − standard error (*n* = 3 biologically independent samples). For photosynthetic parameters, statistical analysis was performed in GraphPad Prism using One-way ANOVA and means were separated with Tukey’s post-test at a significance level of *p* < 0.05 (see the figure legends for details). Error bars represent the standard deviation (s. d.).

## Results and discussion

### Optimization of CRISPR-Cas9 editing constructs

*Nicotiana benthamiana* contains two pairs of homologous *LCYe* and *ZEP* genes: *LCYe1, LCYe2, ZEP1,* and *ZEP2.* In order to simultaneously knock out both homeologs, sgRNAs were designed on regions of sequence conservation among the two homeologs (Fig. S1a). These sgRNAs were cloned in the pDGB3-α1 Agrobacterium transformation vector (Fig. S1b) and used for transient agroinfiltration of 4-weeks-old *N. benthamiana* leaves, in combination with the pAtCas9-NPTII vector, carrying an *Arabidopsis* codon-optimized CAS9 gene (Vazquez-Vilar et al. 2016) under the control of ubiquitin (UBI) promoter. The pHsCAS9 vector, with a human codon-optimized CAS9 gene under the control of the CaMV 35S promoter (Vazquez-Vilar et al. 2016) was used as a control (Fig. S1b). After 6 days, DNA was isolated and analyzed by amplicon sequencing on an Illumina MiSeq platform. The results (Table S2) showed that leaves agroinfiltrated with the *Arabidopsis* codon-optimized CAS9 exhibited significant (3–6%) Indel frequencies at the target sites, and negligible (< 0.15%) frequencies at the non-target ones, while the human codon-optimized *Cas9* was much less efficient in our experimental conditions. Based on these results, pDGB3-α1- ZEP_T1, pDGB3-α1- LCYe_T5 and pAtCas9-NPTII vectors were chosen for stable combinatorial transformation.

### Combinatorial genome editing and direct LC–MS screening for zeaxanthin accumulation

Stable combinatorial transformation was performed as described in “Materials and methods”. 32 regenerated plantlets were obtained from four different transformation experiments, of which 25 survived being transferred to soil. After two weeks of adaptation, DNA was extracted for PCR screening, using specific primers for the presence of the vectors containing *sgRNA-LCYe T5, sgRNA-ZEP T1* and the *CAS9* transgene (Table S1). The different vector combinations recovered are summarized in Table 1. 8/25 independent T_0_ plants (32%) contained all three constructs, indicating a very high co-transformation efficiency. This observation is consistent with previous reports (Li and Durbin 2009), and can be interpreted in the sense that only a subpopulation of plant cells is competent for transformation, and that each cell in this subpopulation can be transformed by multiple *Agrobacterium* cells. While co-transformation using direct DNA transfer methods is widely used (Zhu et al. 2008), *Agrobacterium*-mediated stable co-transformation is much less used and has never, to our knowledge, applied in *N. benthamiana.*

**Table 1:** PCR screening of transformed T_0_ *N. benthamiana* plants for the presence of the vectors containing CAS9 (pAtCas9-NPTII) and the vectors containing the two selected sgRNAs, targeting *LCYe* (pDGB3-α 1-LCYe-T5) and *ZEP * genes (pDGB3- α1-ZEP-T1)

Transgene	N. T_0_ plants
*CAS9*	9
*CAS9*, *sgRNA-LCYe*	5
*CAS9*, *sgRNA-ZEP*	3
*CAS9*, *sgRNA-LCYe*, *sgRNA-ZEP*	8

Primers used for PCR are listed in Table S1

Given the high editing efficiency obtained in transient transformation experiments, we decided to screen the T_1_ progeny directly for accumulation of zeaxanthin. Since, in our chromatographic system, zeaxanthin co-migrates with chlorophyll (Chl*a*) (Fig. S2) we used a semi-quantitative liquid chromatography-mass spectrometry (LC–MS) method to discriminate between the two molecules (see “Materials and methods”). T_1_ progenies from two independent T_0_ triple transformants were screened for the presence of zeaxanthin. The results (Table S3) showed that for the L9 line, 20 out of 22 T_1_ progeny plants accumulated zeaxanthin as the only xanthophyll, while 2 accumulated trace amounts of lutein; for the L11 line, all 18 T_1_ progeny plants showed completed disappearance of lutein and neoxanthin, and 3 accumulated zeaxanthin and β-carotene as the only carotenoids. This carotenoid composition is reminiscent of that of primitive unicellular algal taxa, such as the Glaucophyta and the Cyanidiophyceae (Sandmann 2021).

Next, we scored the presence of the *CAS9* transgene and the gene editing efficiencies in a subset of the same lines by PCR amplification using appropriate primers. 10 out of 26 analyzed lines had lost the *CAS9* transgene. Amplicons containing the target sites were subjected to Sanger sequencing and the data were analyzed using the ICE software to predict the knockout (KO) score (Table S3) (for details, see Materials and methods). Almost all T_1_ progeny plants showed high (> 90) KO scores for *LCYe1*, *LCYe2* and *ZEP2* genes, while 11 out of 26, all derived from T_0_ line 9, exhibited medium–low KO score for *ZEP1,* indicating that ZEP1 could be still partially functional.

Based on these results, two lines (hereinafter named HZ (high zeaxanthin) -9 and -11) were finally selected, based on the following criteria: accumulation of zeaxanthin as the only xanthophyll, high KO scores for *LCYe1*, *LCYe2* and *ZEP2* genes, medium–low (HZ-9) or high (HZ-11) KO score for the *ZEP1* gene, and absence of the *CAS9* transgene.

### Genomic and phenotypic characterization of the HZ-9 and HZ-11 mutants

To confirm the genotype of the HZ-9 and HZ-11 plants and the absence of off-target mutations, we subjected them to whole genome short read (2 × 150 bp) resequencing. The reads were aligned on a newly available chromosome-level *N. benthamiana* genome assembly (Ranawaka et al. 2023) and indels were scored on both on-target and off-target sites carrying up to three mismatches (Table S4). The HZ-11 line carried homozygous frameshift mutations in the *LCYe2,* and *ZEP2* genes, while for the *LCYe1* and *ZEP1* genes, diallelic frameshift mutations (− 1/1 and 1/− 4 respectively) were identified. In all cases, the mutations resulted in premature termination of the predicted proteins (Fig. S3). HZ-9 also carried homozygous frameshift mutations in the *LCYe1*, *LCYe2,* and *ZEP2* genes, resulting in premature termination of the predicted proteins, while for *ZEP1* an in-frame homozygous deletion (− 9/− 9) was identified, resulting in a 3-amino acid deletion in the predicted protein that is localized outside the ZEP catalytic domain (Kim et al. 2018) (Fig. S3). This 3-amino acid deletion is consistent with the lower KO score exhibited by the ZEP1 gene in this mutant, as well as with the less severe phenotypes observed. For the off-target loci, there are no relevant differences between the mutants and WT (Table S4). Both HZ lines and the WT carried homozygous 1 bp indels in three different off-target intergenic regions, probably due to sequence differences between our laboratory strain and the strain used for the generation of the chromosome-level assembly (Ranawaka et al. 2023).

**Fig. 2:**
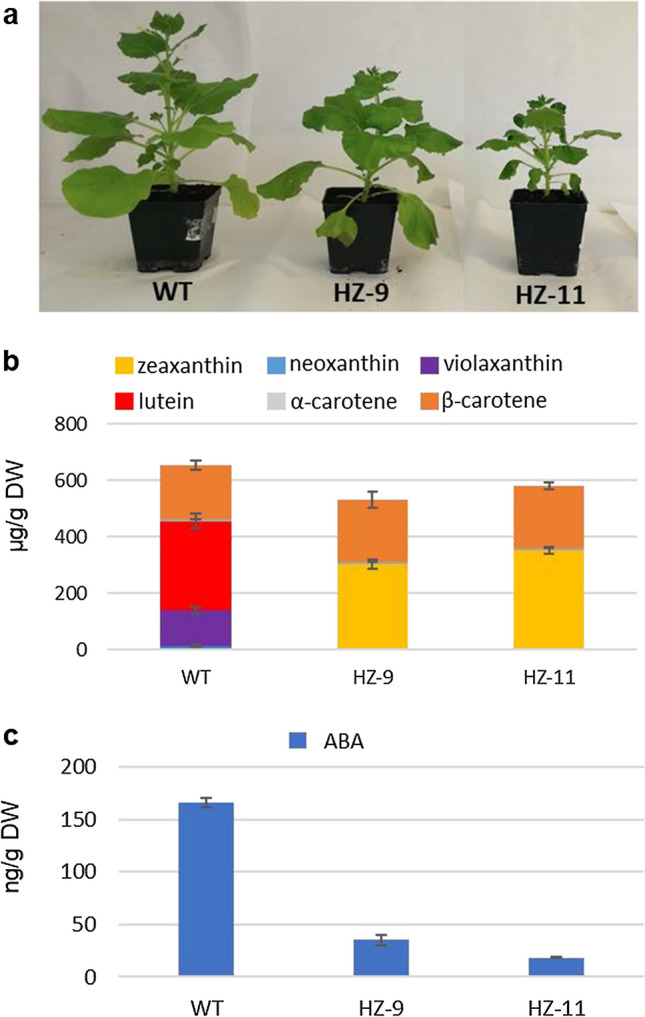
Phenotypic characterization. **a** Phenotypes of 6 weeks old WT and HZ plants. **b** Carotenoid and **c** ABA contents of leaves from WT and HZ lines. Data are the average ± stdev of three biological replicates. Quantitative data can be found in Table S5

Under our growth conditions (16 h light/8 h dark, 150 µmol photons m^−2^ s^−1^, 24 °C) the T_2_ HZ-9 and HZ-11 plants showed a phenotypic gradient with respect to WT, with HZ-9 being slightly stunted, and HZ-11 more evidently so (Fig. 2a). To get an accurate quantification of xanthophyll levels in leaves of WT and HZ lines, we performed HPLC–PDA analyses on saponified and non-saponified leaf extracts from T_2_ plants (see “Materials and methods”) (Fig. S2). The results (Fig. 2b, Table S5) indicate that (i) in both the HZ-9 and HZ-11 plants the xanthophylls violaxanthin, neoxanthin and lutein are completely substituted by zeaxanthin; (ii) HZ-11 has a higher zeaxanthin content within the edited lines. Both edited T_2_ *CAS9*-free plants were confirmed to accumulate mostly zeaxanthin and β-carotene. Zeaxanthin was on average 303.3 and 350.3 µg g^−1^ of Dry Weight (DW) in HZ-9 (*LCY1 LCY2 ZEP2*) and HZ-11 (*LCY1 LCY2 ZEP1 ZEP2*), respectively, and was not detectable in WT plants. Total carotenoids were decreased in HZ-9 and HZ-11 (19% and 11%, respectively) compared to WT (Table S5).

Abscisic acid (ABA) is a well-described hormone regulating several aspects of plant physiology, such as reducing water loss in leaves under drought conditions (Cutler et al. 2010; Lee and Luan 2012). ABA is synthesized starting from 9-*cis*-epoxycarotenoids that are produced from zeaxanthin by ZEP, then cleaved to the C15 intermediate xanthoxin by the enzyme 9-*cis*-epoxycarotenoid dioxygenase (NCED) (Schwartz et al. 1997), and finally converted to ABA via ABA-aldehyde. In *Arabidopsis*, which has only one *ZEP* gene, *ZEP* mutants have severely impaired ABA levels (Rock and Zeevaart 1991). We investigated the ABA content of HZ-9 and HZ-11 lines by LC–MS (see “Materials and methods”). Both lines showed significant decreases in ABA levels (79% and 89%, respectively with respect to the WT; Fig. 2c, Table S5) with the quadruple HZ-11 mutant showing the most severe phenotype. This suggests that in *N. benthamiana*, both *ZEP* genes contribute to ABA biosynthesis and that the bulk of the leaf ABA content is synthesized through 9-*cis*-epoxycarotenoid precursors, rather than through the recently discovered ZEP-independent pathway (Jia et al. 2022). The ABA and carotenoid deficiency was reflected in a retarded growth with respect to the WT, especially noticeable in HZ-11 (Fig. 2a).

### Photosynthetic complex characterization

For photosynthetic characterization, T_2_ progeny plants of the two mutants were grown at 23 °C, under an 8 h light/16 h dark photoperiod at 150 µmol photons m^−2^ s^−1^, with ABA exogenous supplementation (Fig. S4) (see “Materials and methods”). Under these conditions, both HZ-9 and HZ-11 plants showed reduced (− 44%) Chl content per leaf area compared with WT plants (Table 2).

**Table 2:** Pigment content and photosynthetic parameters, determined for leaves of WT and HZ lines

	WT	HZ-9	HZ-11
µg Chl/cm^2^	37.14 ± 8.00^a^	19.21 ± 2.17^b^	19.52 ± 3.63^b^
Chl *a*/*b*	3.42 ± 0.12^a^	3.76 ± 0.12^b^	3.71 ± 0.14^b^
Chl/Car	3.48 ± 0.13^a^	3.48 ± 0.12^a^	3.52 ± 0.08^a^
*F*_*v*_/*F*_*m*_	0.82 ± 0.01^a^	0.76 ± 0.01^b^	0.76 ± 0.01^b^
*F*_0_ (normalized to leaf Chl content)	0.71 ± 0.02^a^	0.59 ± 0.01^b^	0.58 ± 0.02^b^
*F*_max_ (normalized to leaf Chl content)	4.13 ± 0.12^a^	2.50 ± 0.07^b^	2.47 ± 0132^b^
Total photoxidizable PSI (Δ*A*_max_ 830–875 nm, a.u.)	1.16 ± 0.07^a^	0.87 ± 0.04^a^	0.87 ± 0.09^a^
PSII antenna size (*t*_2/3_^−1^ · 10^–3^, ms^−1^)	2.54 ± 0.06^a^	2.48 ± 0.06^a^	2.54 ± 0.06^a^
PSI antenna size	3.63 ± 0.66^a^	2.80 ± 0.62^b^	2.91 ± 0.58^b^

Chl/Car, molar ratio between chlorophylls (*a* + *b*) and carotenoids; Chl *a/b*, molar ratio between Chls *a* and *b*. Values of *F*_0_ and *F*_max_ (minimal and maximal Chl fluorescence of PSII, respectively) were normalized to the corresponding Chl content per unit leaf surface (µg Chls cm^−2^). Total photoxidizable PSI (PSI leaf content, Δ*A*_max_) and PS antenna size were measured as reported in the methods. All data are expressed as mean ± s.d., *n* = 5 biologically independent plants. Values marked with different letters are significantly different from each other within the lane (ANOVA, followed by Tukey’s post-hoctest at a significance level of *P* < 0.05)

Chl and carotenoid content, and PSII/PSI functionality were measured in WT and HZ leaves. The Chl *a/b* ratio was significantly higher in both edited plants, suggesting reduced light-harvesting complexes (LHCs) content and/or altered PSI/PSII ratio, while the Chls/Carotenoid ratio was not affected. HZ plants showed *F*_*V*_/*F*_*M*_ ratios (= (*F*_*M*_–*F*_0_)/*F*_*M*_, i.e. the maximal photochemical yield of PSII) of 0.76 vs. 0.82 for the WT (Maxwell and Johnson 2000). This reduction was ascribed to a marked decrease in *F*_*M*_ (− 40% than WT value), which is consistent with a well-known fluorescence quenching due to zeaxanthin bound to the LHCs (Formaggio et al. 2001).

PSI efficiency (ΔAmax, i.e. maximum photoxidizable PSI/leaf surface) was reduced in the mutant compared with the WT, suggesting that xanthophyll composition might affect either PSI activity and/or accumulation of the complex. We then quantified the functional antenna cross-section of both PSs, by Chl fluorescence induction in the presence of the inhibitor DCMU for PSII, and by P700 oxidation kinetic for PSI (see details in the Methods section). PSII antenna size was similar in all genotypes, implying that the light-harvesting function was not significantly reduced in the absence of lutein, violaxanthin, and neoxanthin; in contrast, PSI functional antenna was slightly smaller (− 20%) in HZ lines than in WT (Table 2).

**Fig. 3:**
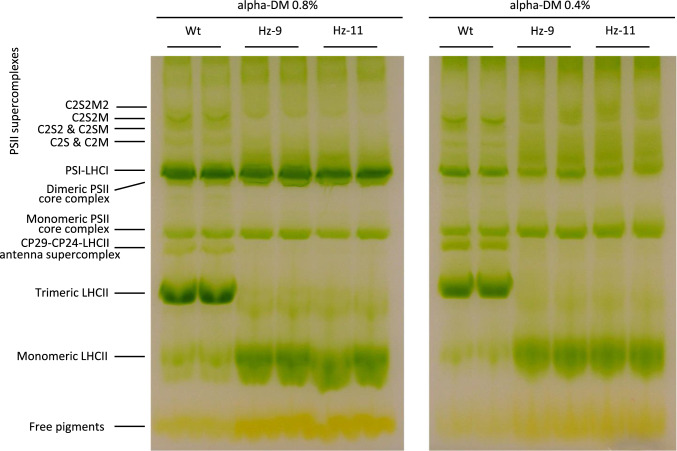
Organization of thylakoid pigment–protein complexes. Thylakoid pigment–protein complexes from WT and HZ lines were separated by non-denaturing Deriphat–PAGE following solubilization of membranes with either  0.8% α-DM (*left panel*) or 0.4% α-DM (*right panel*). Thylakoids (35 μg of Chls) were loaded in each lane. Tentative attribution of PSII-LHCII supercomplex bands is based on Caffarri S. et al. EMBO J. 2009

Separation of pigment-protein complexes by nondenaturing Deriphat-PAGE, upon solubilization of thylakoids with the mild detergent α-DM 0.8%, showed different organization of photosystems in edited lines compared to WT (Fig. 3). In detail, we observed a complete absence of trimeric LHCII and of the LHCII-CP29-CP24 antenna supercomplex in HZ lines, while the band of monomeric LHCs was much more represented than in WT. Most of the high MW PSII supercomplexes were missing in the HZ lines. Altered stability of the LHCII trimeric state is consistent with that reported in other *Arabidopsis* xanthophyll mutants, e.g. *aba* and *lut2* (Hurry et al. 1997; Lokstein et al. 2002) and confirms that trimerization ability is weakened whenever the xanthophyll composition is altered. Consistently, a lower abundance of PSII supercomplexes were found upon thylakoid solubilization. In contrast, the PSI-LHCI band was detected in all genotypes, indicating the two PSs do not have the same sensitivity to the perturbation in xanthophyll composition, in agreement with the phenotype of the *Arabidopsis npq2lut2* mutant (Havaux et al. 2004). Interestingly, treatment of membranes with a lower detergent concentration (α-DM 0.4% *w*/*v*, Fig. 3) was not enough to efficiently solubilize the PSI-LHCI supercomplex, however, it resulted in lower disassembly of the high MW PSII supercomplexes, which became detectable even in HZ thylakoids, although separation suffered for broadening of protein bands.

Both native PAGE and antenna size measurements revealed maintenance of functional Lhca and Lhcb folded subunits in genotypes with altered xanthophyll contents, as well as similar Car contents of purified LHCs (Fig. S5). These results confirmed that xanthophyll composition of LHC is flexible (Carbonera et al. 2022), as previously shown by LHCs purified from *Arabidopsis* carotenoid mutants (Fuciman et al. 2012). Recently, transplastomic tobacco plants containing astaxanthin as the only carotenoid have been produced and shown to possess functional PSI and PSII, as well as capacity to perform NPQ (Xu et al. 2020).

Immunotitration of photosynthetic subunits in thylakoid membranes confirmed a lower PSI/PSII ratio in the mutant, and a reduced biochemical antenna size of PSII mainly due to a lower content of LHCII (Lhcb1 is the major subunit of the trimeric LHCII), while abundance of the monomeric antenna Lhcb4 appears unaffected by change in xanthophyll composition. The other major components of the photosynthetic electron transport chain (ATPase and Cytochrome b6) maintained the same stoichiometry with PSII in all genotypes (Fig. S6, Table S6). It is worth noting that a far lower LHCII abundance, a reduced trimer stability, and a consequent decrease in PSII-LHCII supercomplex abundance, did not impair PSII functional antenna size in HZ lines. Rather, constitutive zeaxanthin accumulation only reduced PSI antenna cross-section (Fig. S7, Table 2). This seemingly contradictory evidence can be explained by hypothesizing LHCII depletion mainly involved stroma-exposed domains of thylakoids, rather than grana partitions. LHCII-S and LHCII-M trimers form the PSII supercomplexes and are stably retained in the grana partitions; while mobile LHCII-L trimers get relocated between grana and stromal domains through reversible phosphorylation events (Galka et al. 2012) to dynamically modulate the antenna size of photosystems thus fine-tuning excitation pressure on them. Besides the association of one P-LHCII trimer with PSI, several reports showed that (i) unphosphorylated LHCII-L populate stromal domains and function as a PSI antenna (Bressan et al. 2018; Bos et al. 2019) and (ii) more than one LHCII-L increase the PSI absorption cross section in WT plants (Bos et al. 2017). In *Arabidopsis* mutants with altered PSI conformations, LHCII docking sites are impaired and LHCII-L are retained in the grana even upon phosphorylation, thus suggesting a molecular recognition mechanism is active in determining LHCII re-distribution between grana and stromal domains (Bressan et al. 2018; Schiphorst et al. 2022). PSI-LHCI and monomeric LHCII binding zeaxanthin only could reasonably have impaired interactions. Recent results (Bykowski et al. 2021) showed that mutations in the carotenoid biosynthetic pathway led to morphological aberrations at the thylakoid level and changed fluidity of the photosynthetic membranes—both phenomena being expected to impact on LHC relative distribution between thylakoid domains. Finally, it cannot be excluded that monomerization of LHCII in HZ lines might impair the stability of this antenna in the stromal-exposed regions, resulting in pauperization of LHCII in this domain and in a reduced PSI antenna cross-section. This is consistent with the report (Yang et al. 1998) that monomeric LHCII is the target of proteolytic degradation, which occurs during photo-acclimation upon migration of antenna to the stroma-exposed domains of thylakoids, where proteases are located. As regards PSII, we cannot rule out that zeaxanthin-LHCII can assemble into trimers in grana domains, even considering the protein density in stacked grana is very high (Kirchhoff 2008); this would promote LHCII-LHCII contacts even in HZ plants, and possibly excitation energy transfers which result in a negligible reduction of antenna cross-section. These functional contacts would be less stable than in WT plants, thus more prone to monomerization upon detergent solubilization of membranes (Fig. 3).

### Non-photochemical quenching

**Fig. 4:**
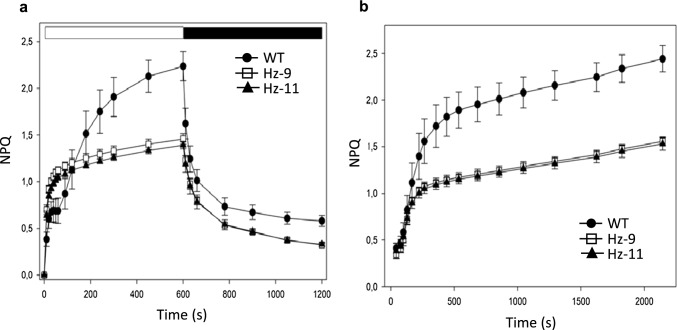
Analysis of NPQ kinetics. **a** Kinetics of the formation and relaxation of photoprotective energy dissipation (total NPQ), measured on leaves at 1200 µmol photons m^–2^ s^–1^ at RT. **b** Kinetics of NPQ measured along a light intensity curve (42→2144 µmol photons m^–2^ s^–1^). Leaves were given 5 min of illumination at 150 µmol photons m^–2^s^–1^ before starting the light curve, to fully activate the Calvin Cycle. Total NPQ was measured at stepwise (2 min) increasing light intensities (see also Fig. S4A) in order to obtain a light response curve, which gives insights into the excitation energy quenching capacity and the current light adaptation state of the genotypes. Data are expressed as mean ± stdev, *n* = 5 biologically independent leaves

Kinetics of the formation and relaxation of photoprotective energy dissipation (total NPQ) were measured on leaves at 1200 µmol photons m^−2^ s^−1^, 23 °C. The induction of NPQ in the mutants showed a rapid phase during the first 100 s, resulting in a faster rise of quenching than observed in the WT, reaching saturation at an NPQ level of 1.4 vs. 2.3 in WT upon 10 min of actinic light. Dark relaxation of NPQ was significantly slower in HZ plants, in agreement with previous results with *Arabidopsis* mutant binding zeaxanthin in their antenna systems (Havaux et al. 2004)(Fig. 4a). NPQ kinetics measured along a light intensity curve (42 → 2144 µmol photons m^−2^ s^−1^, see also Fig. S8) showed a lower dissipative capacity in both edited lines than in WT (Fig. 4b). The reduced NPQ activity in mutant plants can be ascribed to the differential effect of HZ mutation on the quenching processes (namely qE, qZ, qI) which contribute to the overall Chl fluorescence quenching (Nilkens et al. 2010). The rapidly reversible quenching qE is strictly dependent on both lumen acidification and PsbS but also requires zeaxanthin biosynthesis. Thus, a faster NPQ rise was detected in HZ lines since zeaxanthin was already bound to their LHCs at the onset of illumination, while a slower rise of NPQ in WT plants can be ascribed to the limitation by zeaxanthin synthesis. In addition, a zeaxanthin-dependent NPQ component called qZ, formed within 10–15 min of illumination, was reported as independent of lumen acidification (Nilkens et al. 2010). We assume a complete qZ was already developed in dark-adapted HZ plants. This is consistent with a fluorescence quenching in LHCs that resulted from zeaxanthin *vs.* violaxanthin binding to the inner allosteric site L2 (Formaggio et al. 2001) and involved in the formation of a PsbS-/pH-independent quenching mechanism (Dall'Osto et al. 2005). The reduced growth of HZ lines in low light, even when supplemented with exogenous ABA, is consistent with sustained energy dissipation, deriving from a lower fluorescence yield, in dark-adapted plants. Photoinhibitory processes contribute to residual quenching qI, which reverts at a longer time scale (> 30 min). At the end of the dark relaxation, the extent of qI was significantly lower in HZ lines (Fig. 4), thus suggesting these genotypes have a full capacity to counteract photoinhibition.

**Fig. 5:**
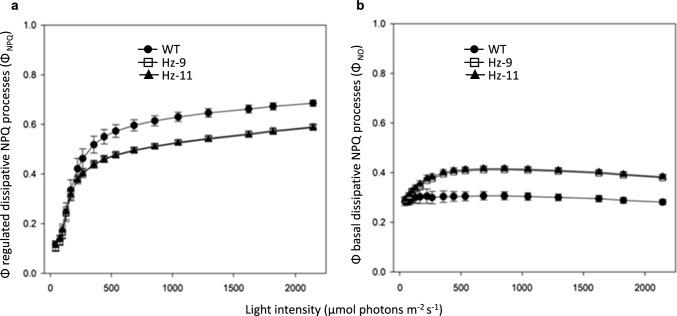
Effects of changing light intensities on the relationships among Φ_NPQ_ and Φ_NO_. Quantum yield of the non-photochemical processes that are capable of quenching Chl excited states, namely regulated energy quenching (or Φ_NPQ_, panel **a**) and constitutive (basal or Φ_NO_, panel **b**) dissipative processes, were measured along a light intensity curve (42→2144 µmol photons m^–2^ s^–1^) as described in Figure S4A. The regulated processes are triggered as a response to light in contrast to the second type of processes involving passively energy dissipation. Data are expressed as mean ± stdev, *n* = 5 biologically independent leaves

Chl florescence analysis allowed to calculate additional quenching parameters, namely Φ_NPQ_ and Φ_NO_, which represent the fraction of excitation energy discarded through the two competing non-photochemical routes, namely that induced by feedback downregulated events (ΔpH-dependent NPQ response) and that representing passively, light-independent excitation energy losses (Kramer et al. 2004). In HZ leaves, the quantum yield of basal dissipative processes was slightly higher, thus being consistent with a higher thermal energy dissipation within the antenna system due to constitutive zeaxanthin binding (Dall'Osto et al. 2005; Gilmore and Ball 2000), providing a “background” dissipation at all light intensities. Consequently, being the fraction of energy absorbed by the PSII and used in photochemistry (Φ_PSII_) the same in all genotypes at all irradiances tested (Fig. S8), a matching decrease in the regulated dissipation was observed in HZ plants since both types of non-photochemical mechanisms represent competing dissipative channels (Fig. 5) (Niyogi et al. 1998).

**Fig. 6:**
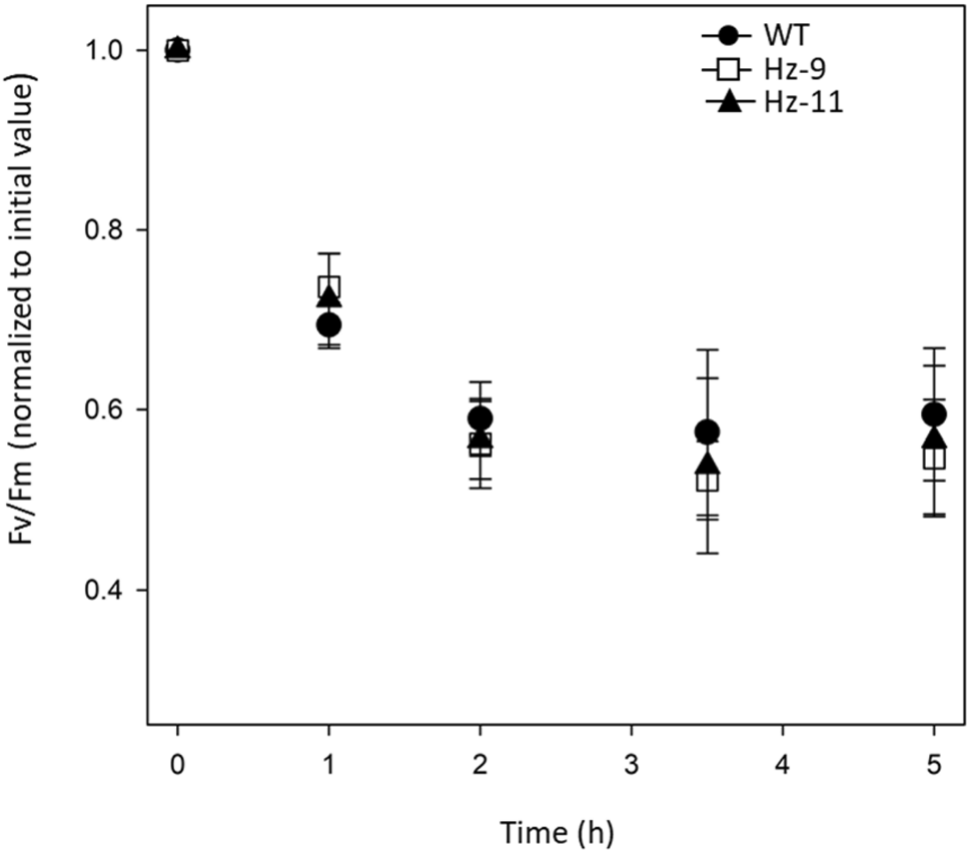
Photoinhibition of leaves exposed to excess light. PSII photoinhibition (Fv/Fm decay) was followed in WT and HZ lines, treated at 2000 µmol photons m^–2^ s^–1^, 23 °C for 5 h. Data are expressed as mean ± stdev, *n* = 5 biologically independent samples. Values are not significantly different from each other within the same time point (ANOVA followed by Tukey’s post-hoc test at a significance level of *P* < 0.05), indicating that PSII binding only zeaxanthin and *β*-carotene is fully able to limit photoinhibition

We then investigated the photoresistance of HZ lines: leaf discs were exposed to excess light (2000 µmol photons m^−2^ s^−1^, 23 °C for 5 h), a condition effective for the induction of oxidative stress and photoinhibition. Indeed, this treatment promotes a rapid decrease in PSII quantum efficiency as measured by the Chl fluorescence parameter *F*_*V*_/*F*_*M*_. Results (Fig. 6) showed that the extent of PSII photoinhibition was essentially the same in WT and HZ lines. The high phototolerance of HZ plants cannot be explained by their PS function (Table 2): (i) the quantum yield of PSII photochemistry measured at increasing light intensities was similar in WT and HZ plants (Fig. S8), even at the irradiance used for the excess light stress test (Fig. 6); (ii) the maximum photoxidizable PSI per leaf surface was reduced in HZ lines as compared to WT (Table 2); (iii) the photoprotective thermal energy dissipation via NPQ was also impaired in HZ plants (Fig. 3). The high phototolerance of HZ plants cannot be ascribed to the lower LHCII content, rather antenna depletion would have resulted in higher photosensitivity (Dall'Osto et al. 2010). We can reasonably attribute the photoresistance in HZ plants to the zeaxanthin present in their chloroplasts. Indeed, carotenoid biosynthesis mutants of *Arabidopsis* revealed zeaxanthin has the highest antioxidant capacity among xanthophylls (Havaux et al. 2007). Moreover, a significant reduction in Φ_NPQ_ in HZ lines (Fig. 5) suggested LHC components in mutants were less efficient than WT-LHC in thermally deactivating Chl first singlet excited state, the precursor species to highly reactive singlet oxygen. However, zeaxanthin binding to LHC subunits resulted in a compensative increase in Φ_NO_, which estimates the energy flux via the process of constitutive energy dissipation (Hendrickson et al. 2004). Such strengthened dissipative channel likely enhanced photoresistance in HZ lines by limiting the release of singlet oxygen (Dall'Osto et al. 2012).

## Conclusions

*Nicotiana benthamiana* is a model system for plant transformation and is used as a platform for the production of biopharmaceuticals. In this study, we describe an efficient combinatorial genome editing approach of *N. benthamiana*, designed to accumulate a normally not accumulated endogenous xanthophyll such as zeaxanthin. Using optimized genome editing constructs through combinatorial transformation with *A. tumefaciens*, and selecting plants using both genomic and phenotypic characterization (by LC/MS and LC/PDA), our approach efficiently allows the achievement of stably transformed Cas9-free *N. benthamiana* T_2_ plants accumulating zeaxanthin as the only xanthophyll. Within the two independent edited lines, HZ-9 (*LCYe1 LCYe2 ZEP2*) showed both *LCYe* genes and the *ZEP2* gene inactivated, while HZ-11 (*LCYe1 LCYe2 ZEP1 ZEP2*) showed all four genes were inactivated. Edited plants underwent a compensatory increase in PSII/PSI ratios and a loss of the large-size PSII supercomplexes, while the level of PSI-LHCI supercomplex was unaffected. They also exhibited reduced activity of the photoprotective mechanism NPQ, while PSII photoinhibition was similar for all genotypes upon exposure to excess light, consistent with the antioxidant and photoprotective role of zeaxanthin in vivo.

### Supplementary Information

Below is the link to the electronic supplementary material.

**File S1** Gene IDs (TXT 1 KB)

**File S2** Vector sequences (TXT 60 KB)

**File S3** p19.gb map (GB 12 KB)

**File S4** pAtCas9-NPTII.gb map (GB 20 KB)

**File S5** pHsCAS9.gb map (GB 17 KB)

**File S6** pSgRNA.gb map (GB 10 KB)

Fig. S1 Design of vectors for simultaneous CRISPR-CAS9 editing of the homeologous *LCYe1-LCYe2* and *ZEP1-ZEP2 N. benthamiana* genes. Fig. S2 LC-PDA analysis of leaf pigments before and after saponification. Fig. S3 Alignment of CDS sequence of wild-type and edited *LCYe* and *ZEP* genes deduced by whole genome re-sequencing (a) and schematic representation of wild-type and edited Lcye and Zep proteins generated by CRISPR-Cas9 (b). Fig. S4 WT and HZ *N. benthamiana* plants, grown for 4 weeks under an 8 h light/16 h dark photoperiod at 100 µmol photons m^−2^ s^−1^, with and without ABA exogenous supplementation. Fig. S5 Sucrose density gradient fractionation of WT and HZ solubilized thylakoids, and carotenoid composition of LHC-containing bands. Fig. S6 Quantification of major photosynthetic subunits in WT and mutant thylakoids. Fig. S7 Functional antenna size of photosystems. Fig. S8 Analysis of PSII quantum yield during photosynthesis. (a) The light gradient used. (b) Kinetics of Φ_PSII_, which reflects the fraction of absorbed photons used to drive PSII photochemistry (PPTX 8767 KB)

Table S1 Primers used for PCR or sequencing. Table S2 Frequency of the target (*LCY, ZEP*) and off-target InDels by agroinfiltration of *N. benthamiana* leaves and NGS analysis. Table S3 Percentage of carotenoid distribution measured by LC/APCI/MS, *CAS9* presence and gene KO scores in two T_1_ families transformed with the three constructs. Table S4 Whole genome resequencing of the HZ-9 and HZ-11 mutants. Table S5 (a) Carotenoid (µg/g DW) and ABA (ng/g DW) contents of T_2_ progenies derived from HZ-9 and HZ-11. Asteriks indicate significant variations in an ANOVA plus Tukey's t-test between HZ lines and WT (yellow: *p* ≤ 0.01). (b) Gene KO scores in the same lines, calculated with ICE. Table S6 Immunotitration of photosynthetic subunits in thylakoid membranes. Amounts of Chl loaded: 0.15–0.30–0.45 µg/each genotype. The abundance of each subunit in the mutant is expressed as a percentage of the corresponding content in the wild-type. Data are expressed as mean ± stdev, n = 3. Values marked with different letters are significantly different from each other within the lane (ANOVA, followed by Tukey’s post test at a significance level of *p* < 0.05). (XLSX 40 KB)

## Data Availability

WGS data has been deposited in the Sequence Read Archive (SRA) under Bioproject PRJNA975873.

## Abbreviations

ABA: Abscisic acid
LCYe: Lycopene epsilon cyclase
ZEP: Zeaxanthin epoxidase
LHCI: Light-harvesting complex I
LHCII: Light-harvesting complex II
PSI: Photosystem I
PSII: Photosystem I
NPQ: Nonphotochemical quenching
AMD: Age-related macular degeneration
HZ: High-Zeaxanthin
CRISPR: Clustered Regularly Interspaced Short Palindromic Repeats
CAS9: Crispr-Associated Protein 9
LC: Liquid chromatography
HRMS: High-Resolution Mass Spectrometry
APCI: Atmospheric Pressure Chemical Ionization (APCI)
HESI: Heated ElectroSpray Ionization
PDA: PhotoDiode Array
LC–MS: Liquid Chromatography-Mass Spectrometry
Chl: Chlorophyll
