# Supplementary material for: Generation and physiological characterization of genome-edited Nicotiana benthamiana plants containing zeaxanthin as the only leaf xanthophyll
Source: Planta. 2023 Oct 5;258(5):93. doi: 10.1007/s00425-023-04248-3 (PMC10556183; doi:10.1007/s00425-023-04248-3)
Supplement: Supplementary file 7 — Fig. S1 Design of vectors for simultaneous CRISPR-CAS9 editing of the homeologous LCYe1-LCYe2 and ZEP1-ZEP2 N. benthamiana genes. Fig. S2 LC-PDA analysis of leaf pigments before and after saponification. Fig. S3 Alignment of CDS sequence of wild-type and edited LCYe and ZEP genes deduced by whole genome re-sequencing (a) and schematic representation of wild-type and edited Lcye and Zep proteins generated by CRISPR-Cas9 (b). Fig. S4 WT and HZ N. benthamiana plants, grown for 4 weeks under an 8 h light/16 h dark photoperiod at 100 µmol photons m−2 s−1, with and without ABA exogenous supplementation. Fig. S5 Sucrose density gradient fractionation of WT and HZ solubilized thylakoids, and carotenoid composition of LHC-containing bands. Fig. S6 Quantification of major photosynthetic subunits in WT and mutant thylakoids. Fig. S7 Functional antenna size of photosystems. Fig. S8 Analysis of PSII quantum yield during photosynthesis. (a) The light gradient used. (b) Kinetics of ΦPSII, which reflects the fraction of absorbed photons used to drive PSII photochemistry (PPTX 8767 KB) [file 425_2023_4248_MOESM7_ESM.pptx]

## Slide 1
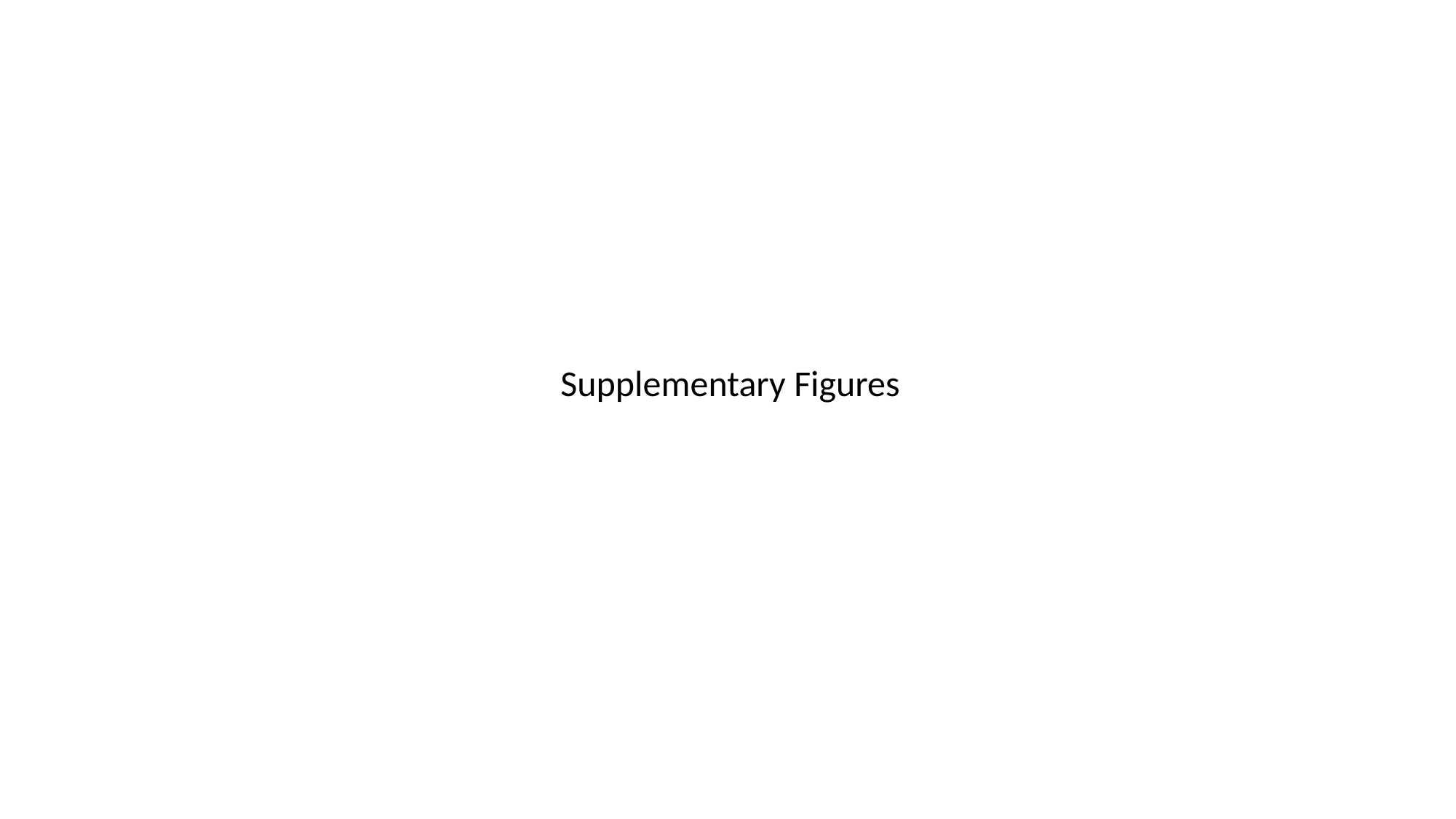

Supplementary Figures

## Slide 2
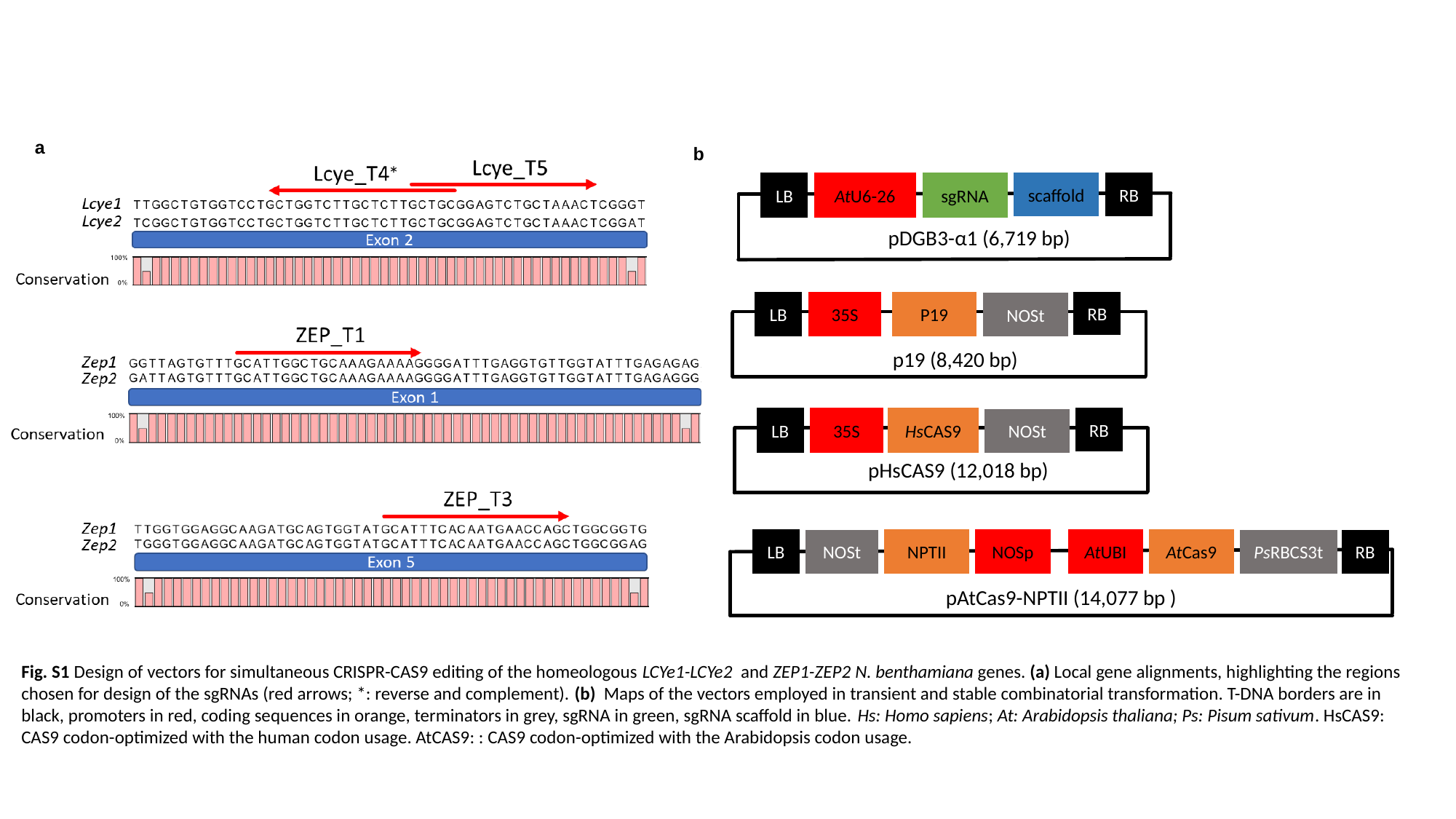

a
b
*
LB
AtU6-26
sgRNA
scaffold
RB
pDGB3-α1 (6,719 bp)
LB
35S
P19
RB
NOSt
p19 (8,420 bp)
LB
35S
HsCAS9
RB
NOSt
pHsCAS9 (12,018 bp)
LB
NPTII
NOSp
AtUBI
AtCas9
NOSt
PsRBCS3t
RB
pAtCas9-NPTII (14,077 bp )
Fig. S1 Design of vectors for simultaneous CRISPR-CAS9 editing of the homeologous LCYe1-LCYe2  and ZEP1-ZEP2 N. benthamiana genes. (a) Local gene alignments, highlighting the regions chosen for design of the sgRNAs (red arrows; *: reverse and complement). (b)  Maps of the vectors employed in transient and stable combinatorial transformation. T-DNA borders are in black, promoters in red, coding sequences in orange, terminators in grey, sgRNA in green, sgRNA scaffold in blue. Hs: Homo sapiens; At: Arabidopsis thaliana; Ps: Pisum sativum. HsCAS9: CAS9 codon-optimized with the human codon usage. AtCAS9: : CAS9 codon-optimized with the Arabidopsis codon usage.

## Slide 3
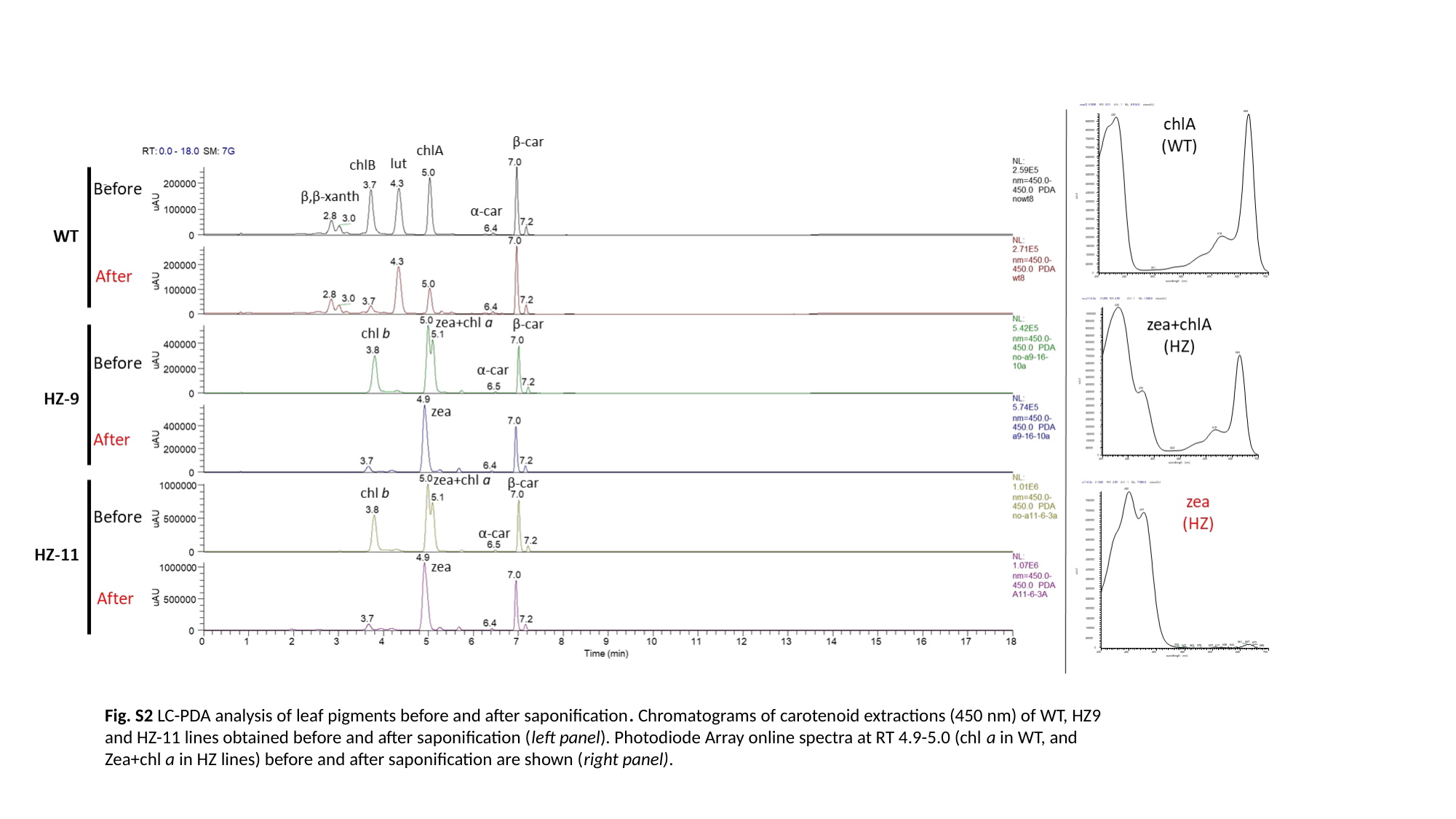

Fig. S2 LC-PDA analysis of leaf pigments before and after saponification. Chromatograms of carotenoid extractions (450 nm) of WT, HZ9 and HZ-11 lines obtained before and after saponification (left panel). Photodiode Array online spectra at RT 4.9-5.0 (chl a in WT, and Zea+chl a in HZ lines) before and after saponification are shown (right panel).

## Slide 4
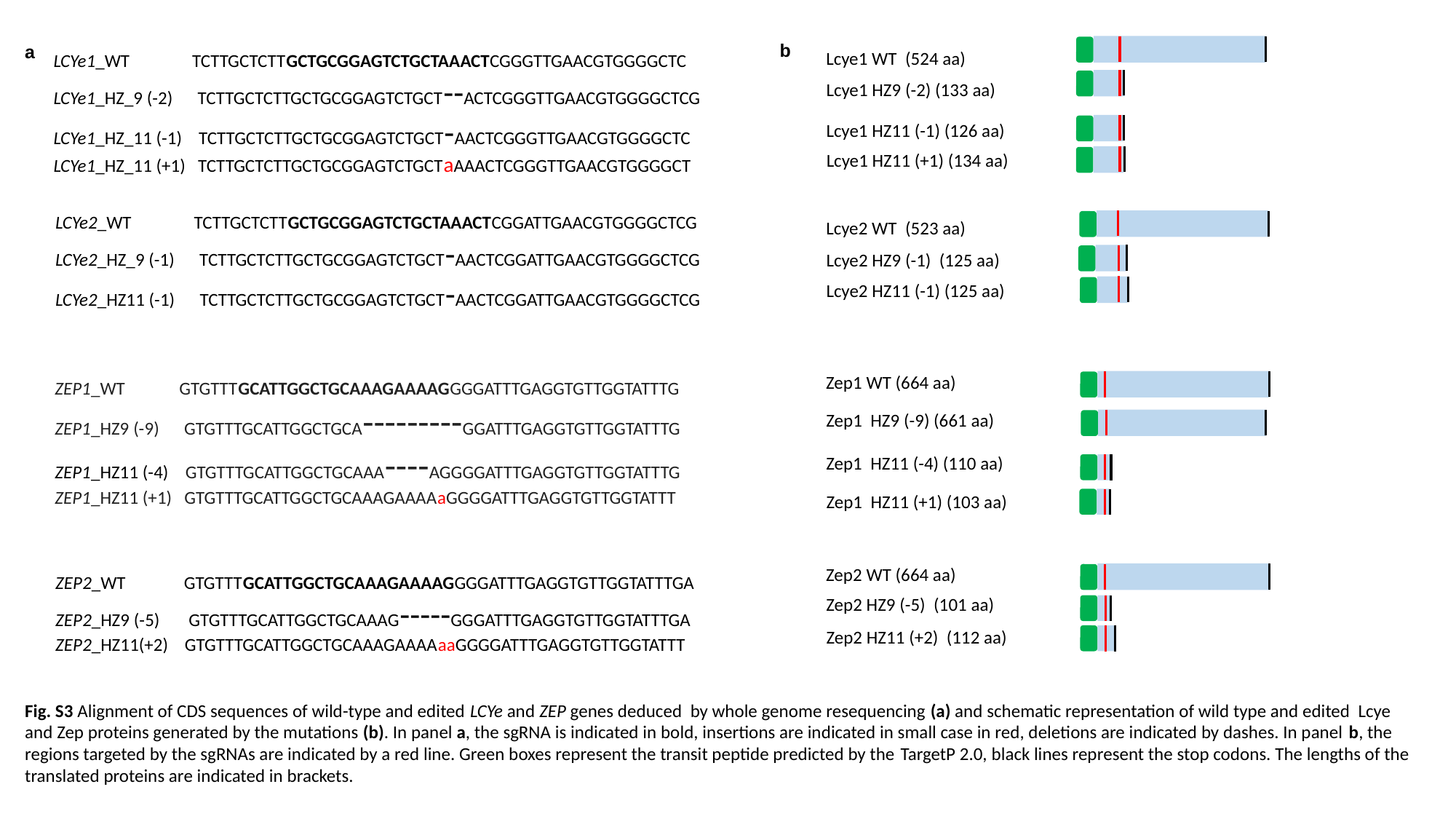

b
a
Lcye1 WT (524 aa)
LCYe1_WT TCTTGCTCTTGCTGCGGAGTCTGCTAAACTCGGGTTGAACGTGGGGCTC
LCYe1_HZ_9 (-2) TCTTGCTCTTGCTGCGGAGTCTGCT--ACTCGGGTTGAACGTGGGGCTCG
LCYe1_HZ_11 (-1) TCTTGCTCTTGCTGCGGAGTCTGCT-AACTCGGGTTGAACGTGGGGCTC
LCYe1_HZ_11 (+1) TCTTGCTCTTGCTGCGGAGTCTGCTaAAACTCGGGTTGAACGTGGGGCT
Lcye1 HZ9 (-2) (133 aa)
Lcye1 HZ11 (-1) (126 aa)
Lcye1 HZ11 (+1) (134 aa)
LCYe2_WT TCTTGCTCTTGCTGCGGAGTCTGCTAAACTCGGATTGAACGTGGGGCTCG
LCYe2_HZ_9 (-1) TCTTGCTCTTGCTGCGGAGTCTGCT-AACTCGGATTGAACGTGGGGCTCG
LCYe2_HZ11 (-1) TCTTGCTCTTGCTGCGGAGTCTGCT-AACTCGGATTGAACGTGGGGCTCG
Lcye2 WT (523 aa)
Lcye2 HZ9 (-1) (125 aa)
Lcye2 HZ11 (-1) (125 aa)
Zep1 WT (664 aa)
ZEP1_WT GTGTTTGCATTGGCTGCAAAGAAAAGGGGATTTGAGGTGTTGGTATTTG
ZEP1_HZ9 (-9) GTGTTTGCATTGGCTGCA---------GGATTTGAGGTGTTGGTATTTG
ZEP1_HZ11 (-4) GTGTTTGCATTGGCTGCAAA----AGGGGATTTGAGGTGTTGGTATTTG
ZEP1_HZ11 (+1) GTGTTTGCATTGGCTGCAAAGAAAAaGGGGATTTGAGGTGTTGGTATTT
Zep1 HZ9 (-9) (661 aa)
Zep1 HZ11 (-4) (110 aa)
Zep1 HZ11 (+1) (103 aa)
Zep2 WT (664 aa)
ZEP2_WT GTGTTTGCATTGGCTGCAAAGAAAAGGGGATTTGAGGTGTTGGTATTTGA
ZEP2_HZ9 (-5) GTGTTTGCATTGGCTGCAAAG-----GGGATTTGAGGTGTTGGTATTTGA
ZEP2_HZ11(+2) GTGTTTGCATTGGCTGCAAAGAAAAaaGGGGATTTGAGGTGTTGGTATTT
Zep2 HZ9 (-5) (101 aa)
Zep2 HZ11 (+2) (112 aa)
Fig. S3 Alignment of CDS sequences of wild-type and edited LCYe and ZEP genes deduced by whole genome resequencing (a) and schematic representation of wild type and edited Lcye and Zep proteins generated by the mutations (b). In panel a, the sgRNA is indicated in bold, insertions are indicated in small case in red, deletions are indicated by dashes. In panel b, the regions targeted by the sgRNAs are indicated by a red line. Green boxes represent the transit peptide predicted by the TargetP 2.0, black lines represent the stop codons. The lengths of the translated proteins are indicated in brackets.

## Slide 5
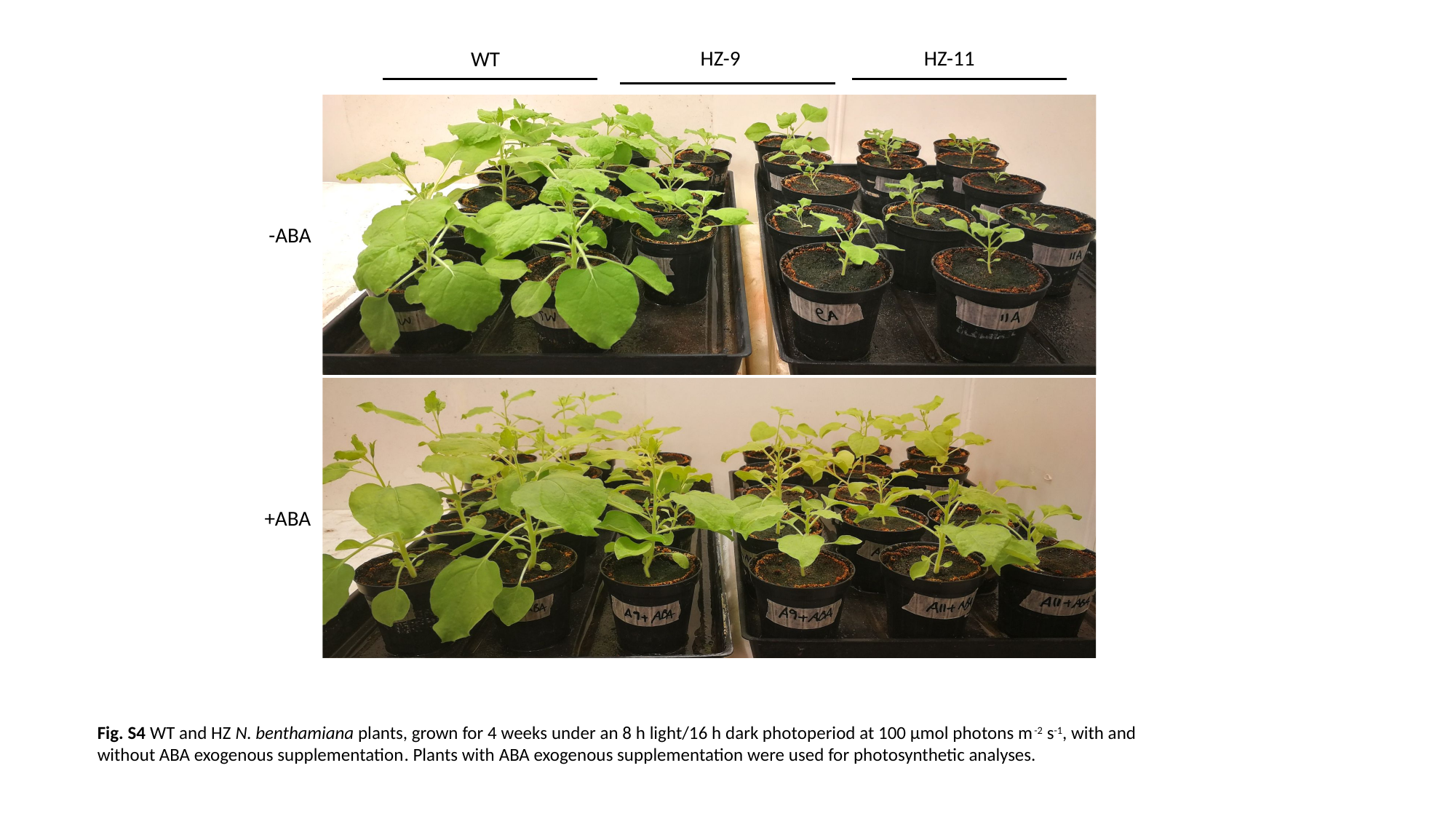

HZ-9
HZ-11
WT
-ABA
+ABA
Fig. S4 WT and HZ N. benthamiana plants, grown for 4 weeks under an 8 h light/16 h dark photoperiod at 100 µmol photons m-2 s-1, with and without ABA exogenous supplementation. Plants with ABA exogenous supplementation were used for photosynthetic analyses.

## Slide 6
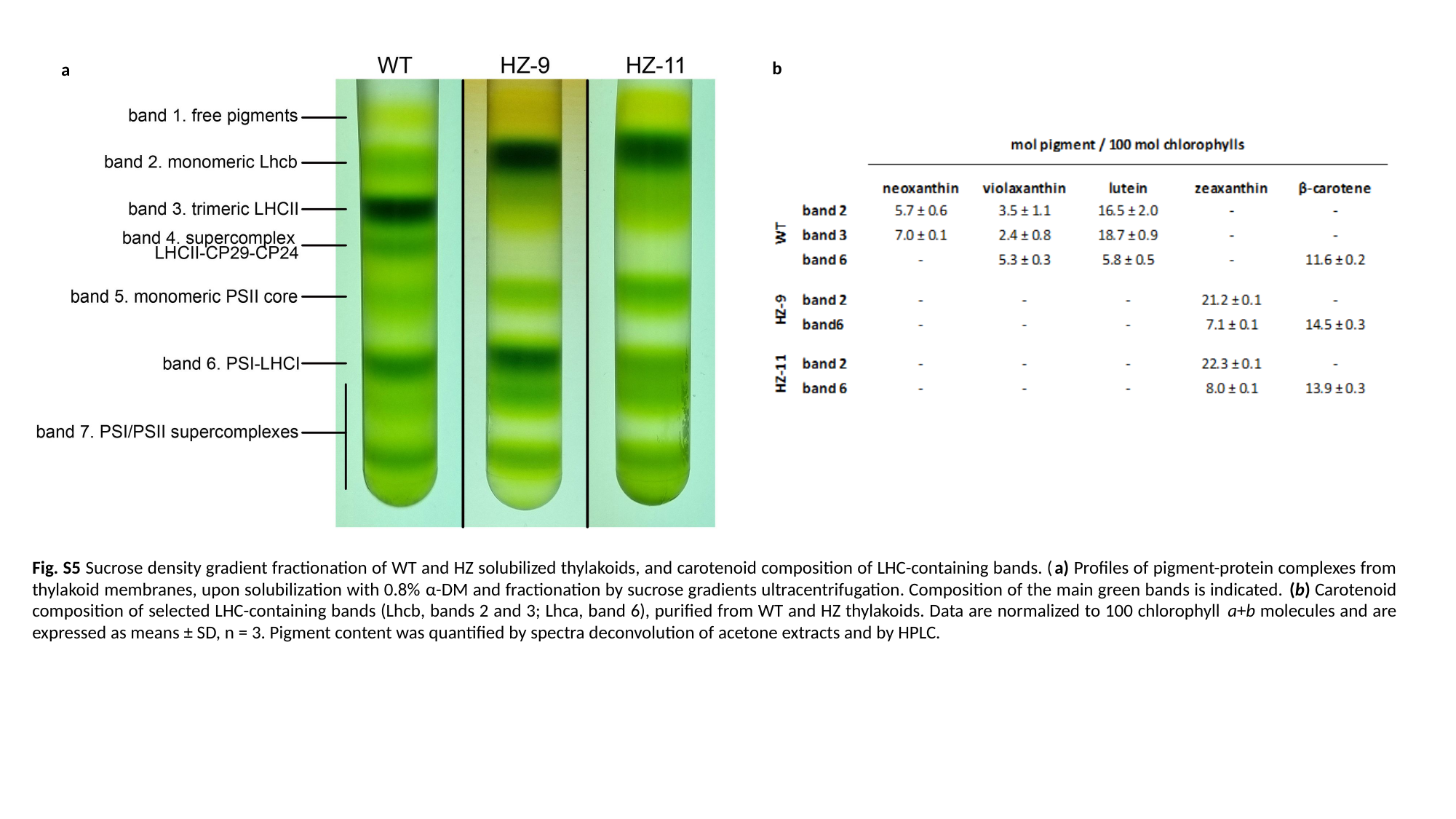

b
a
Fig. S5 Sucrose density gradient fractionation of WT and HZ solubilized thylakoids, and carotenoid composition of LHC-containing bands. (a) Profiles of pigment-protein complexes from thylakoid membranes, upon solubilization with 0.8% α-DM and fractionation by sucrose gradients ultracentrifugation. Composition of the main green bands is indicated. (b) Carotenoid composition of selected LHC-containing bands (Lhcb, bands 2 and 3; Lhca, band 6), purified from WT and HZ thylakoids. Data are normalized to 100 chlorophyll a+b molecules and are expressed as means ± SD, n = 3. Pigment content was quantified by spectra deconvolution of acetone extracts and by HPLC.

## Slide 7
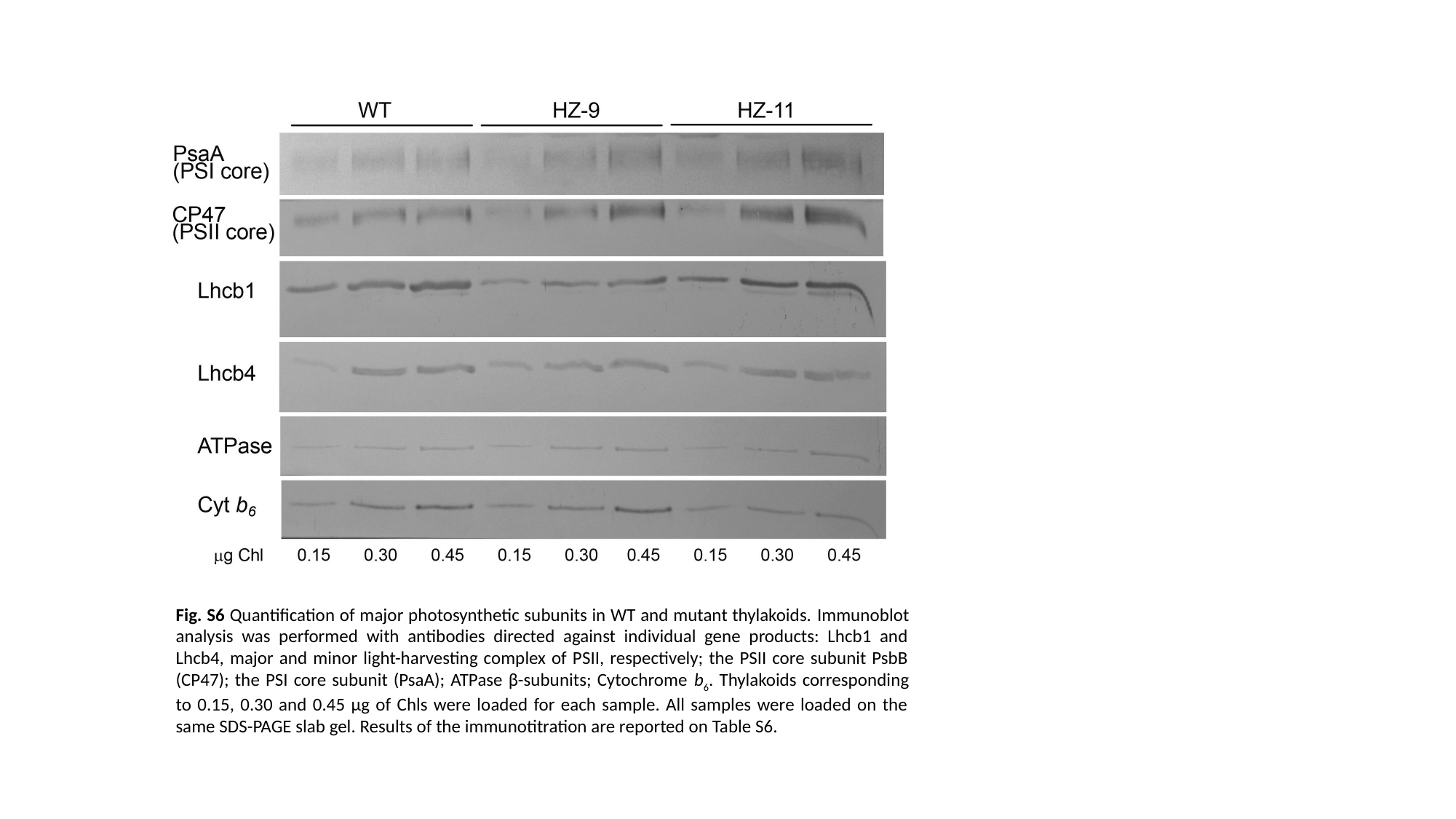

Fig. S6 Quantification of major photosynthetic subunits in WT and mutant thylakoids. Immunoblot analysis was performed with antibodies directed against individual gene products: Lhcb1 and Lhcb4, major and minor light-harvesting complex of PSII, respectively; the PSII core subunit PsbB (CP47); the PSI core subunit (PsaA); ATPase β-subunits; Cytochrome b6. Thylakoids corresponding to 0.15, 0.30 and 0.45 μg of Chls were loaded for each sample. All samples were loaded on the same SDS-PAGE slab gel. Results of the immunotitration are reported on Table S6.

## Slide 8
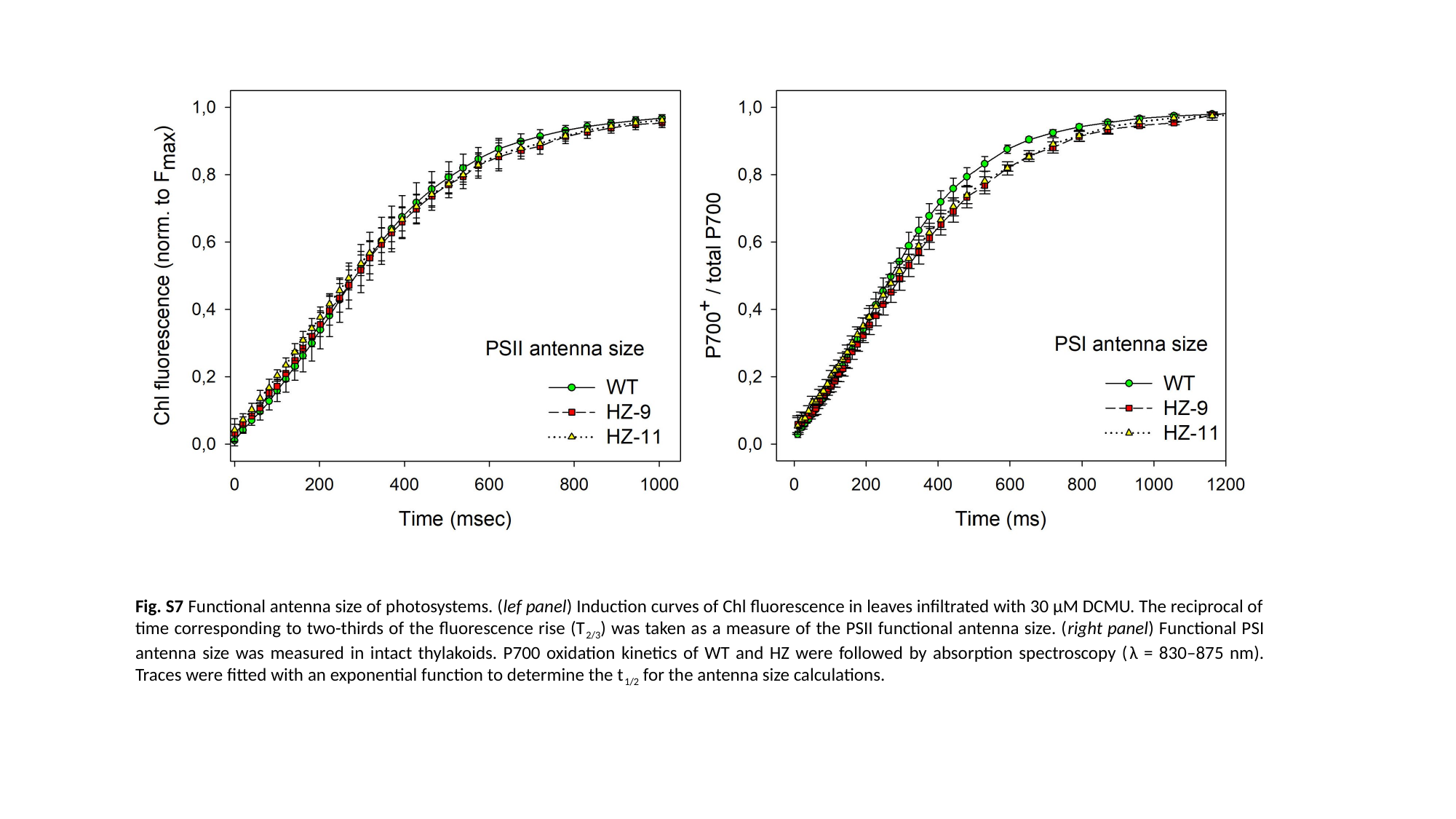

Fig. S7 Functional antenna size of photosystems. (lef panel) Induction curves of Chl fluorescence in leaves infiltrated with 30 µM DCMU. The reciprocal of time corresponding to two-thirds of the fluorescence rise (T2/3) was taken as a measure of the PSII functional antenna size. (right panel) Functional PSI antenna size was measured in intact thylakoids. P700 oxidation kinetics of WT and HZ were followed by absorption spectroscopy (λ = 830–875 nm). Traces were fitted with an exponential function to determine the t1/2 for the antenna size calculations.

## Slide 9
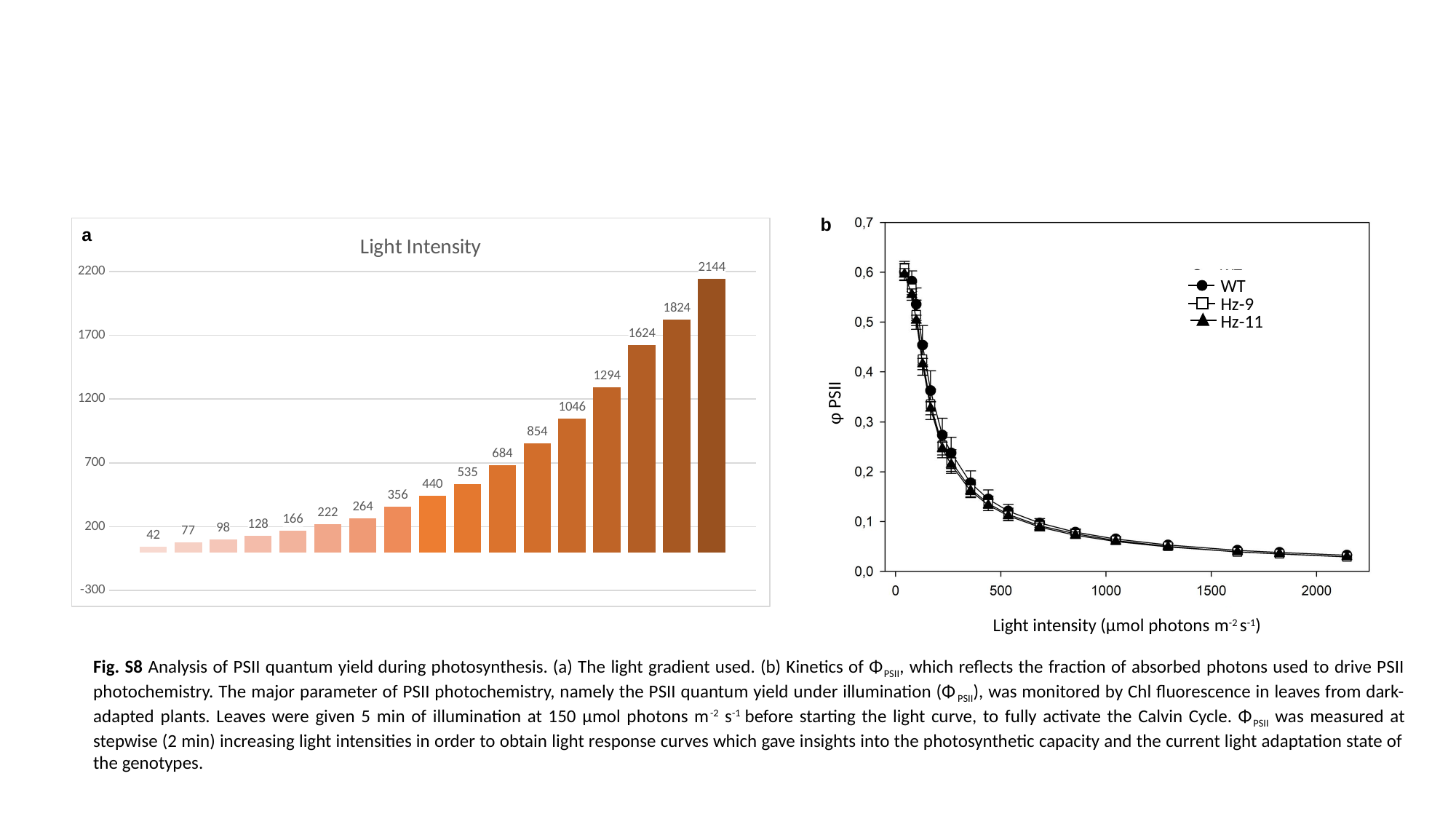

b
### Chart: Light Intensity
| Category | | | | | | | | | | | | | | | | | |
|---|---|---|---|---|---|---|---|---|---|---|---|---|---|---|---|---|---|a
WT
Hz-9
Hz-11
 φ PSII
Light intensity (µmol photons m-2 s-1)
Fig. S8 Analysis of PSII quantum yield during photosynthesis. (a) The light gradient used. (b) Kinetics of ΦPSII, which reflects the fraction of absorbed photons used to drive PSII photochemistry. The major parameter of PSII photochemistry, namely the PSII quantum yield under illumination (ΦPSII), was monitored by Chl fluorescence in leaves from dark-adapted plants. Leaves were given 5 min of illumination at 150 µmol photons m-2 s-1 before starting the light curve, to fully activate the Calvin Cycle. ΦPSII was measured at stepwise (2 min) increasing light intensities in order to obtain light response curves which gave insights into the photosynthetic capacity and the current light adaptation state of the genotypes.
